# Chemical Constituents from the Roots of *Angelica reflexa* That Improve Glucose-Stimulated Insulin Secretion by Regulating Pancreatic β-Cell Metabolism

**DOI:** 10.3390/pharmaceutics15041239

**Published:** 2023-04-13

**Authors:** Hyo-Seon Kim, Dahae Lee, Young-Hye Seo, Seung-Mok Ryu, A-Yeong Lee, Byeong-Cheol Moon, Wook-Jin Kim, Ki-Sung Kang, Jun Lee

**Affiliations:** 1 Herbal Medicine Resources Research Center, Korea Institute of Oriental Medicine (KIOM), Naju 58245, Republic of Korea; 2 Cooperative-Center of Natural Product Central Bank for Biological Evaluation, College of Korean Medicine, Gachon University, Seongnam 13120, Republic of Korea

**Keywords:** *Angelica reflexa*, *Ostericum koreanum*, glucose-stimulated insulin secretion, diabetes, hyperglycemia, marmesinin

## Abstract

The aim of this study was to discover bioactive constituents of *Angelica reflexa* that improve glucose-stimulated insulin secretion (GSIS) in pancreatic β-cells. Herein, three new compounds, namely, koseonolin A (**1**), koseonolin B (**2**), and isohydroxylomatin (**3**), along with 28 compounds (**4**–**31**) were isolated from the roots of *A*. *reflexa* by chromatographic methods. The chemical structures of new compounds (**1**–**3**) were elucidated through spectroscopic/spectrometric methods such as NMR and HRESIMS. In particular, the absolute configuration of the new compounds (**1** and **3**) was performed by electronic circular dichroism (ECD) studies. The effects of the root extract of *A*. *reflexa* (KH2E) and isolated compounds (**1**–**31**) on GSIS were detected by GSIS assay, ADP/ATP ratio assay, and Western blot assay. We observed that KH2E enhanced GSIS. Among the compounds **1**–**31**, isohydroxylomatin (**3**), (−)-marmesin (**17**), and marmesinin (**19**) increased GSIS. In particular, marmesinin (**19**) was the most effective; this effect was superior to treatment with gliclazide. GSI values were: 13.21 ± 0.12 and 7.02 ± 0.32 for marmesinin (**19**) and gliclazide at a same concentration of 10 μM, respectively. Gliclazide is often performed in patients with type 2 diabetes (T2D). KH2E and marmesinin (**19**) enhanced the protein expressions associated with pancreatic β-cell metabolism such as peroxisome proliferator-activated receptor γ, pancreatic and duodenal homeobox 1, and insulin receptor substrate-2. The effect of marmesinin (**19**) on GSIS was improved by an L-type Ca^2+^ channel agonist and K+ channel blocker and was inhibited by an L-type Ca^2+^ channel blocker and K^+^ channel activator. Marmesinin (**19**) may improve hyperglycemia by enhancing GSIS in pancreatic β-cells. Thus, marmesinin (**19**) may have potential use in developing novel anti-T2D therapy. These findings promote the potential application of marmesinin (**19**) toward the management of hyperglycemia in T2D.

## 1. Introduction

Type 2 diabetes (T2D) is a metabolic disorder characterized by a relative lack of insulin secretion by pancreatic β cells, which interferes the adipocyte lipolysis, skeletal muscle glucose uptake, and glucose metabolism in liver. Its result is postprandial hyperglycemia, which must be controlled to prevent T2D [1]. In many therapeutic approaches to reduce postprandial hyperglycemia in T2D, the ability of pancreatic β cells to secrete insulin has been evaluated as important [2]. Among hypoglycemic oral drugs, gliclazide as a second-generation sulfonylurea augments the ability to secrete insulin from pancreatic β-cells in the treatment of T2D [3], but a major side effect in using gliclazide is weight gain [4]. Therefore, the search for anti-T2D drugs with few drawbacks has been attracting attention, with interest shifting to naturally derived compounds, as they are known to possess less toxic side effects [5].

The plant material used in this study, called Ganghwal in Korea, is one of the most important perennials native to Asian countries belonging to the family Umbelliferae [6]. In the Korean Pharmacopoeia, the origin of Ganghwal is defined as the root of *Ostericum koreanum* Maxim., or the rhizome and root of *Notopterygium incisum* Ting or *Notopterygium forbesii* Boissier (Umbelliferae). Its roots have long been used in the treatment of the common cold, headache, neuralgia, and arthralgia in the Chinese Herbal Medicine [7]. In Korea, the root of this plant has been widely used as herbal medicine to treat coldness, pain, and dampness in the body [8]. Ganghwal, currently cultivated and distributed in Korea, has been used as the colloquial name of *Ostericum koreanum* (Maxim.) Kitag., but it is considered to also refer to *Ostericum grosseserratum* (Maxim.) Kitag. Further, there is the viewpoint that Ganghwal is actually the same species as *Ostericum praeteritum* Kitag. or *Angelica genuflexa* Nutt. ex Torr. & A. Gray, so there has been confusion about the scientific name of this plant [9]. In a recent study on the origin of Ganghwal distributed in Korea, this plant was identified as a new species and was newly named *Angelica reflexa* B.Y.Lee [8,9]. Since there has been much confusion about the origin and scientific name of this plant, it is necessary to establish new research results on the components and efficacy of *A*. *reflexa* that have been accurately identified.

Previous biological studies have reported that extracts of this plant have antitumor [10], antibacterial, antimicrobial [11], anti-inflammatory [12], antioxidant [13], acaricidal [7], vasorelaxant [14], and antiasthmatic activities [15]. Several chemical components with biological activities, such as essential oil [16,17], coumarins [18], chromones [13], phenolics [13], quinic acids [13], and benzofurans [19,20], have been reported from this plant. In addition, this plant has been reported to exhibit no toxicity effects in Neuro-2a neuroblastoma cells (2.5–5 µg/mL) and bone marrow macrophages (5–25 µg/mL) [21,22]. Although many studies have been conducted on this plant using both in vitro and in vivo model systems [7,8,9,10,11,12,13,14,15,16,17,18], its efficiency on glucose-stimulated insulin secretion (GSIS) in pancreatic beta cells has not yet been elucidated. Further, it is still unknown which components are the major contributors to insulin secretion.

In the present study, 31 compounds (**1**–**31**), including three new substances (**1**–**3**), were isolated and identified from the roots extract of *A*. *reflexa* (KH2E) via chromatographic methods, and their effects on GSIS and protein expressions linked to GSIS were examined. This study aimed to determine the anti-diabetic effect of *A*. *reflexa* and to identify new bioactive components responsible for the anti-T2D effect of this plant.

## 2. Materials and Methods

### 2.1. General

An Optizen POP spectrophotometer (Mecasys, Daejeon, Republic of Korea) was used to obtain the UV spectra. A P-2000 polarimeter (JASCO, Easton, MD, USA) was used for optical rotation measurements. Electronic circular dichroism (ECD) spectra were measured with a J-1100 spectropolarimeter (JASCO). NMR spectra were acquired on a 500 MHz NMR spectrometer (Bruker, Karlsruhe, Germany) or DD2 600 MHz FT NMR spectrometer (Agilent Technologies, Santa Clara, CA, USA). HRESIMS data were acquired using a Q-TOF micromass spectrometer (Waters, Milford, MA, USA). Flash chromatography was conducted using a Biotage Selekt chromatography system (Biotage, Uppsala, Sweden) with Sfär Silica HC (10, 25, and 100 g, Biotage) and Sfär C_18_ (30, 120, and 240 g, Biotage) prepacked cartridges. TLC was performed using a precoated TLC plates (RP-18 F254S and silica gel 60 F254, Merck, Darmstadt, Germany).

### 2.2. Plant Material

The dried roots of Ganghwal cultivated in Korea were purchased from a commercial supplier (Omniherb Co., Yeongcheon, Republic of Korea) in 2015. The plant material used for this study was accurately identified as *A. reflexa* by expert morphological identification (Dr. Choi Goya, visual and organoleptic examination specialist for Korean medicinal materials appointed by the Ministry of Food and Drug Safety, Cheongju, Republic of Korea) and genetic identification through comparative analysis with our standard plants. Taxonomic keys for *A. reflexa* have been described in previous study [8]. A voucher specimen (no. 2-15-0547) was deposited at the Herbal Medicine Resources Research Center, Korea Institute of Oriental Medicine (KIOM), Naju, Republic of Korea.

### 2.3. Genetic Analysis

The comparative genetic analysis of the herbal medicine samples used in this work was carried out to identify the plant species of herbal materials based on the ITS (Internal Transcribed Spacer) sequences, including *A. reflexa*, *O. grosseserratum*, *N. forbesii*, and *N. incisum* (see the Supplementary Figure S21). The two samples analyzed were randomly selected from dried plant materials used in this study. Genomic DNA was extracted using the DNeasy^®^ Plant Mini Kit (Qiagen, Valencia, CA, USA), and DNA quality and quantity were measured using an ND-1000 UV/Vis spectrophotometer (NanoDrop, Wilmington, DE, USA). The ITS region was amplified using ITS1 (5′-TCC GTA GGT GAA CCT GCG G-3′) and ITS4 (5′-TCC TCC GCT TAT TGA TAT GC-3′) primers [23]. The amplified ITS sequence was analyzed as previously reported by ClustalW using the BioEdit software 7.2.5 (Raleigh, NC, USA) [24].

### 2.4. Extraction and Isolation

The dried root parts of *A*. *reflexa* (500.0 g) were extracted under reflux with 70% EtOH (2 × 5 L) and concentrated under reduced pressure to obtain a crude extract (KH2E, 96.6 g). The extract (69.2 g) was suspended in distilled water (1.0 L) and sequentially partitioned with several organic solvents to give *n*-hexane- (2.7 g), EtOAc- (2.2 g), *n*-BuOH- (5.7 g), and water-soluble extracts (56.3 g). The *n*-hexane extract (2.7 g) was fractionated using a flash chromatography system (FCS) with a 120 g Sfär C_18_ cartridge (water/MeOH, 50:50 to 0:100) to obtain 36 subfractions (F01–F36). F09 (12.1 mg) was separated using three 10 g Sfär Silica HC cartridges (CHCl_3_/ACN, 99:1) to afford compounds **4** (2.5 mg) and **5** (3.5 mg). Compound **8** (32.0 mg) was obtained by crystallization from F12 (170.0 mg). Further purification of fraction F12 (136.0 mg) was subjected to an FCS using a 100 g Sfär Silica HC cartridge (CHCl_3_/acetone, 99:1 to 0:100) to yield five fractions (F1201–F1205). F1202 (102.0 mg) was further fractionated with a 100 g Sfär Silica HC cartridge (CHCl_3_/acetone, 99:1 to 0:100) to give compounds **6** (8.4 mg) and **7** (2.6 mg), along with 10 other subfractions (F120201-120210). Compounds **21** (10.8 mg) and **30** (6.0 mg) were obtained by FCS using two 30 g Sfär C_18_ cartridges (water/ACN, 70:30 to 0:100) and three 10 g Sfär Silica HC cartridges (CHCl_3_/acetone, 98:2 to 0:100) from F120207 (40.0 mg) and F120210 (14.0 mg), respectively. F13 (63.0 mg) was chromatographed on two 25 g Sfär Silica HC cartridges (CHCl_3_/ACN, 99:1 to 90:10) using an FCS to produce compound **13** (24.5 mg). F14 (54.0 mg) was processed by an FCS with three 25 g Sfär Silica HC cartridges (CHCl_3_/ACN, 99:1 to 0:100) to give compounds **28** (7.4 mg), **24** (2.4 mg), **10** (4.0 mg), and **11** (13.6 mg). Separation of compound **16** (27.6 mg) from F16 (53.0 mg) was conducted using two 25 g Sfär Silica HC cartridges (CHCl_3_/ACN, 99:1 to 0:100). F19 (276 mg) was purified with a 100 g Sfär Silica HC cartridge (CHCl_3_/ACN, 99:1 to 0:100) to afford compounds **22** (77.3 mg), **27** (34.6 mg), and 13 subfractions (F1901-F1913). Chromatographic separation of F1912 (7.0 mg) was processed with three 10 g Sfär Silica HC cartridges (CHCl_3_/ACN, 98:2 to 0:100) to give compound **26** (3.7 mg). Compound **9** (32.0 mg) was obtained by crystallization from F21 (49.4 mg). Compound **20** (4.6 mg) was obtained from F22 (57.0 mg) using two 25 g Sfär Silica HC cartridges (CHCl_3_/ACN, 99:1 to 95:5).

The separation of EtOAc extract (2.2 g) was performed with a 240 g Sfär C_18_ cartridge (water/MeOH, 30:70 to 10:90) to yield 25 fractions (F01–F25). F04 (134.8 mg) was purified using a 25 g Sfär Silica HC cartridge (CHCl_3_/MeOH/water, 95:5:0.4 to 70:30:5) to afford compound **31** (11.3 mg). F05 (136.0 mg) was fractionated using two 30 g Sfär C_18_ cartridges (water/ACN, 90:10 to 60:40) to yield seven fractions (F0501–F0507), and compounds **3** (2.4 mg), **18** (6.5 mg), **29** (2.3 mg), and **19** (8.0 mg) from F0505 were obtained using three 10 g Sfär Silica HC cartridges (CHCl_3_/MeOH/water, 95:5:0.3 to 90:10:0.45). Chromatographic separation of F08 (85.3 mg) was carried out using a 25 g Sfär Silica HC cartridge (*n*-hexane/EtOAc, 70:30 to 30:70) to give compounds **23** (3.5 mg) and **17** (8.3 mg). Separation of F13 (203.7 mg) was conducted using a 25 g Sfär Silica HC cartridge (CHCl_3_/ACN, 99:1 to 70:30) to give compounds **14** (2.3 mg), **15** (12.1 mg), and **12** (12.7 mg) and 16 subfractions (F1301–1316). Repeated flash chromatography of F1315 (13 mg) was carried out using three 10 g Sfär Silica HC cartridges (*n*-hexane/EtOAc, 70:30 to 50:50) to produce compound **25** (6.1 mg). Separation of compounds **1** (13.3 mg) and **2** (8.1 mg) from F16 (98.2 mg) was conducted using a 100 g Sfär Silica HC (CHCl_3_/ACN, 99:1 to 50:50) cartridge.

#### 2.4.1. Koseonolin A (1)

Amorphous solid; [α] ^22^_D_ −13 (*c* 0.1, EtOH); UV (MeOH) λmax (log ε) 228 (4.15), 251 (4.10), 259 (4.07), 268 (4.07), 308 (4.01) nm; ECD (c 0.2 mM, MeCN) Δε −4.7 (215), −3.4 (289), +4.6 (336); HRESIMS *m*/*z* 479.1347 [M − H]^−^ (calcd for C_26_H_23_O_9_, 479.1342).

#### 2.4.2. Koseonolin B (2)

Amorphous solid; [α]^22^_D_ 0 (*c* 0.1, EtOH); UV (MeOH) λmax (log ε) 223 (2.59), 243 (3.98), 317 (3.94) nm; HRESIMS *m*/*z* 467.1737 [M − H]^−^ (calcd for C_26_H_27_O_8_, 467.1706).

#### 2.4.3. Isohydroxylomatin (3)

Amorphous solid; [α]^22^_D_ +38 (*c* 0.1, CHCl_3_); UV (MeOH) λmax (log ε) 211 (3.72), 327 (3.41) nm; ECD (c 0.2 mM, MeCN) Δε +3.0 (207), −1.8 (224), +2.5 (323); HRESIMS *m*/*z* 263.0905 [M + H]^+^ (calcd for C_14_H_15_O_5_, 263.0919).

### 2.5. Computational Methods

The calculation of ECD was performed as described previously [25]. Conformer distributions and ECD calculations were performed in Spartan’14 (Wave-function, Inc., Irvine, CA, USA) and Gaussian’09 (Gaussian, Inc., Wallingford, CT, USA), respectively. The conformers were optimized with DFT [B3LYP functional/6-31+G(d,p) basis set], and ECD calculations were performed at the TDDFT (CAM-B3LYP/SVP basis set, CPCM solvent model in ACN).

### 2.6. Cell Culture and Cell Viability Assay

INS-1 pancreatic β-cells were obtained from Biohermes (Shanghai, China). Cells were cultured in Roswell Park Memorial Institute 1640 medium (Cellgro, Manassas, VA, USA) containing 1% P/S, 2 mM L-glutamine, 0.05 mM 2-mercaptoethanol, 11 mM d-glucose, 1 mM sodium pyruvate, 10% FBS, 10 mM HEPES, and 10 mM HEPES with 5% CO_2_ at 37 °C.

Cell viability was assessed using Ez-Cytox cell viability reagent (Daeil Lab Service Co., Seoul, Republic of Korea) with a modified protocol based on previous study [26]. After treatment, Ez-Cytox cell viability reagent was added to each well. After cultivation for 1 h, the absorbance (490 nm) was recorded using a PowerWave XS microplate reader (Bio-Tek Instruments, Winooski, VT, USA).

### 2.7. GSIS Assay and ADP/ATP Ratio Assay

INS-1 cells were treated with samples diluted in a HEPES buffer solution (200 mmol/L, pH 7.4) for 2 h, followed by treatment with glucose (2.8 mM and 16.7 mM) diluted in a HEPES buffer solution for 1 h. Then, GSIS was determined using a rat insulin ELISA kit (Gentaur, Shibayagi Co. Ltd., Gunma, Shibukaw, Japan) with a protocol based on supplier’s instructions. ADP/ATP ratio was determined using an ADP/ATP ratio assay kit (Sigma-Aldrich, St Louis, MO, USA) with a protocol based on supplier’s instructions.

### 2.8. Western Blot Analysis

Western blot was carried out with a modified protocol based on previous study [27]. The membranes were incubated with the relevant primary antibodies (Cell Signaling, Danvers, MA, USA) for 1 h on ice. They were further incubated with horseradish peroxidase-conjugated anti-rabbit secondary antibodies (Cell Signaling) for 1 h on ice.

### 2.9. Statistical Analysis

Statistical significance was performed by one-way analysis of variance (ANOVA), with the Bonferroni correction for multiple comparisons. Statistical significance was set at *p* < 0.05. All analyses were performed by SPSS Statistics ver. 19.0 (SPSS Inc., Chicago, IL, USA).

## 3. Results

### 3.1. Structural Elucidation of Isolated Compounds

**Figure 1:**
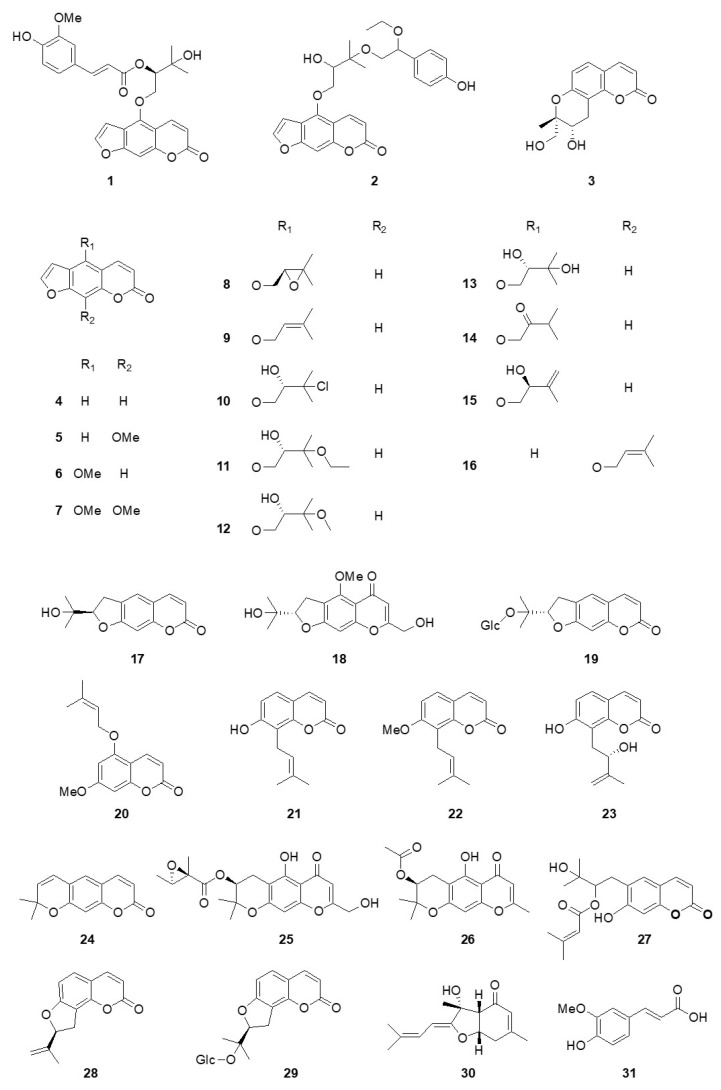
Chemical structures of compounds **1**–**31** from the roots of *A*. *reflexa*.

The chemical structures of the isolated compounds (**1**–**31**) were elucidated through spectroscopic/spectrometric methods such as NMR and HRMS (Figure 1), and in particular, the absolute composition of the new compounds (**1** and **3**) was carried out through ECD studies.

**Figure 2:**
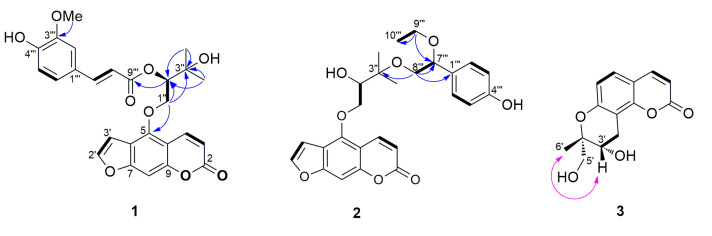
Key COSY (**−**), HMBC (→), and NOESY (↔) correlations of compounds **1**–**3**.

**Figure 3:**
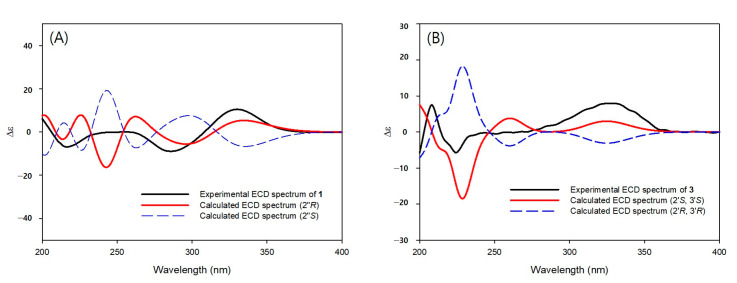
Experimental and calculated ECD spectra of compounds **1** (**A**) and **3** (**B**).

Compound **1** was isolated as an amorphous solid and had a molecular formula of C_26_H_24_O_9_ as determined by HRESIMS (*m*/*z* 479.1347 [M − H]^−^, calcd. C_26_H_23_O_9_). The ^1^H NMR spectrum of **1** showed signals for four doublet and one singlet methines [*δ*_H_ 6.24 (1H, d, *J* = 9.9 Hz, H-3), 7.01 (1H, d, *J* = 2.2 Hz, H-3′), 7.14 (1H, s, H-8), 7.61 (1H, d, *J* = 2.2 Hz, H-2′), and 8.12 (1H, d, *J* = 9.9 Hz, H-4)], indicating the presence of the furanocoumarin skeleton, as well as signals for two methyl groups [*δ*_H_ 1.38 (3H, s, H-4″) and 1.41 (3H, s, H-5″)], a methylene group [*δ*_H_ 4.69 (1H, dd, *J* = 10.2, 8.0 Hz, H-1″) and 4.85 (1H, dd, *J* = 10.2, 2.6 Hz, H-1″)], and an oxygenated methine group [*δ*_H_ 5.44 (1H, dd, *J* = 8.0, 2.6 Hz, H-2″)]. In addition, signals for ABX aromatic spin system [*δ*_H_ 6.94 (1H, d, *J* = 8.2 Hz, H-5′′′), 7.02 (1H, d, *J* = 1.1 Hz, H-2′′′), and 7.08 (1H, dd, *J* = 8.2, 1.3 Hz, H-6′′′)], a *trans*-olefinic group [*δ*_H_ 6.31 (1H, d, *J* = 15.9 Hz, H-8′′′) and 7.67 (1H, d, *J* = 15.9 Hz, H-7′′′)], and a methoxy group [*δ*_H_ 3.95 (3H, s, OMe-3′′′)] were observed in the ^1^H NMR spectrum, which suggested the presence of a ferulic acid moiety (Table 1). The HMBC correlations of H-1″ with C-5 (*δ*_C_ 148.6) /C-2″ (*δ*_C_ 77.4) /C-3″ (*δ*_C_ 71.7) and H-4″/H-5″ with C-2″/C-3″ indicated the presence of oxypeucedanin hydrate [28]. In addition, the downfield shifted H-2″ (*δ*_H_ 5.44) and the HMBC cross peak of H-2″ with C-9′′′ (*δ*_C_ 166.7) indicated that the feruloyl group is linked to oxypeucedanin hydrate C-2″ position (Figure 2). The absolute configurations of C-2″ position of **1** was determined by ECD study. The experimental spectrum was in good agreement with the calculated ECD spectrum of the “*R*“model (Figure 3). Based on this spectroscopic evidence, the structure of compound **1** established and named koseonolin A.

Compound **2** was obtained as an amorphous solid, and had a molecular formula of C_26_H_27_O_8_ as determined by HRESIMS (*m*/*z* 467.1737 [M − H]^−^, calcd. C_26_H_26_O_8_). The ^1^H NMR spectrum of compound **2** was similar to those of **1**, except for the signals of *para*-disubstituted benzene ring [*δ*_H_ 6.80 (1H, d, *J* = 8.2 Hz, H-3′′′/H-5′′′) and 7.19 (1H, d, *J* = 8.3 Hz, H-2′′′/H-6′′′)], ea methyl group [*δ*_H_ 1.18 (3H, t, *J* = 7.0 Hz, H-10′′′)], two oxygenated methylene groups [*δ*_H_ 3.38 (1H, qd, *J* = 9.2, 7.0 Hz, H-9′′′), 3.45 (1H, qd, *J* = 9.2, 7.0 Hz, H-9′′′), 3.52 (1H, dd, *J* = 9.7, 4.9 Hz, H-8′′′), and 3.67 (1H, dd, *J* = 9.7, 6.4 Hz, H-8′′′)], and an oxygenated methine group [*δ*_H_ 4.34 (1H, t, *J* = 5.7 Hz, H-7′′′)] in **2** instead of the ferulic acid moiety in **1** (Table 1). In the HMBC spectrum (Figure 2), the correlations of H-9′′′ with C-7′′′/C-10′′′ and H-8′′′ with C-1′′′/C-7′′′/C-3″ indicated that the 4-(1-ethoxy-2-hydroxyethyl)phenol group is attached on the C-3″ position of oxypeucedanin hydrate. The stereochemistry of **2** was confirmed by using optical rotation value and ECD spectrum. Due to the lack of optical rotation and no Cotton effects in its experimental ECD spectrum, **2** is considered a new racemic mixture. Therefore, the structure of compound **2** was elucidated and named koseonolin B.

Compound **3** was obtained as an amorphous solid and had a molecular formula of C_14_H_14_O_5_ as determined by HRESIMS (*m*/*z* 263.0905 [M + H]^+^, calcd. C_14_H_15_O_5_). Signals for two pairs of doublet methines [*δ*_H_ 6.19 (1H, d, *J* = 9.5 Hz, H-3), 6.78 (1H, d, *J* = 8.3 Hz, H-6), 7.41 (1H, d, *J* = 8.3 Hz, H-5), and 7.86 (1H, d, *J* = 9.5 Hz, H-4)], two methylenes [*δ*_H_ 3.32 (1H, dd, *J* = 16.1, 9.9 Hz, H-4′), 3.43 (1H, dd, *J* = 16.1, 8.0 Hz, H-4′), 3.53 (1H, d, *J* = 10.5 Hz, H-5′), and 3.72 (1H, d, *J* = 10.5 Hz, H-5′)], an oxygenated methine [*δ*_H_ 5.02 (1H, dd, *J* = 9.9, 8.0 Hz, H-3′)], and a methyl group [*δ*_H_ 1.21 (3H, s, H-6′)] were observed in the ^1^H NMR spectrum (Table 1). In addition, 14 carbon signals including 4 unprotonated sp^2^ carbon [*δ*_C_ 114.6 (C-10), 115.4 (C-8), 152.7 (C-9), and 165.8 (C-7)], an oxygenated quaternary carbon [*δ*_C_ 74.6 (C-2′)], and a carbonyl carbon [*δ*_C_ 163.4 (C-2)] were evident from the ^13^C and HSQC NMR spectra. The NMR data of compound **3** were similar to that of previously reported hydroxylomatin [29]. However, the optical rotation value ([α]_D_ +38, *c* 0.1, CHCl_3_) of **3** was different from the those of hydroxylomatin ([α]_D_ -30, *c* 0.2, CHCl_3_). The relative configurations of the C-2′ and C-3′ positions were confirmed at 2′*R,* 3′*R* or 2′*S,* 3′*S* by the NOESY correlation peak of H-3′ with H-6′ (and no correlation peak of H-3′ with H-5′) (Figure 2). The (2′*S,* 3′*S*) absolute configuration of **3** was determined by comparison of the experimental and calculated ECD spectra (Figure 3). Based on this spectroscopic evidence, the structure of compound **3** was established as isohydroxylomatin.

Additionally, 28 known compounds were assigned as psoralen (**4**) [30], xanthotoxin (**5**) [28], bergapten (**6**) [31], isopimpinellin (**7**) [32], (−)-oxypeucedanin (**8**) [33], isoimperatorin (**9**) [31], (−)-saxalin (**10**) [34], (−)-oxypeucedanin hydrate-3”-ethyl ether (**11**) [34], (−)-oxypeucedanin methanolate (**12**) [35], (−)-oxypeucedanin hydrate (**13**) [28], isooxypeucedanin (**14**) [35], pabulenol (**15**) [35,36], imperatorin (**16**) [37], (−)-marmesin (**17**) [28], cimifugin (**18**) [38], marmesinin (**19**) [39], 7-hydroxy-5-[(3-methylbut-2-en-1-yl)oxy]-2H-chromen-2-one (**20**) [40], osthenol (**21**) [18], osthole (**22**) [41], demethylauraptenol (**23**) [42], xanthyletin (**24**) [43,44], alsaticol (**25**) [45], 3′-*O*-acetylhamaudol (**26**) [46], grandivitinol (**27**) [47], angenomalin (**28**) [48], columbianetin *β*-d-glucopyranoside (**29**) [39,49], bisabolagelone (**30**), and *trans*-ferulic acid (**31**) [50].

### 3.2. Effect of KH2E and Compounds **1**–**31** on GSIS

**Figure 4:**
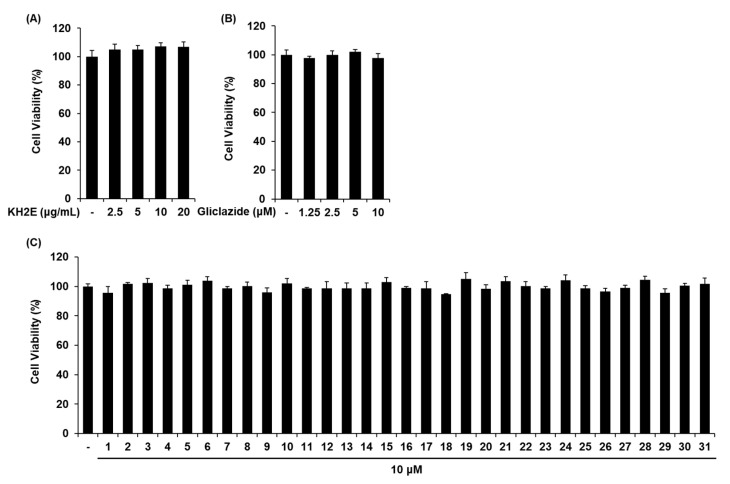
Effects of KH2E and compounds **1**–**31** on cell viability in INS-1 cells. Results of MTT assay for viability of INS-1 cells after 24 h of treatment with (**A**) KH2E, (**B**) gliclazide, and (**C**) compounds **1**–**31** when compared with the untreated control. Data represent the mean ± standard error of the mean (S.E.M.), n = 3.

**Figure 5:**
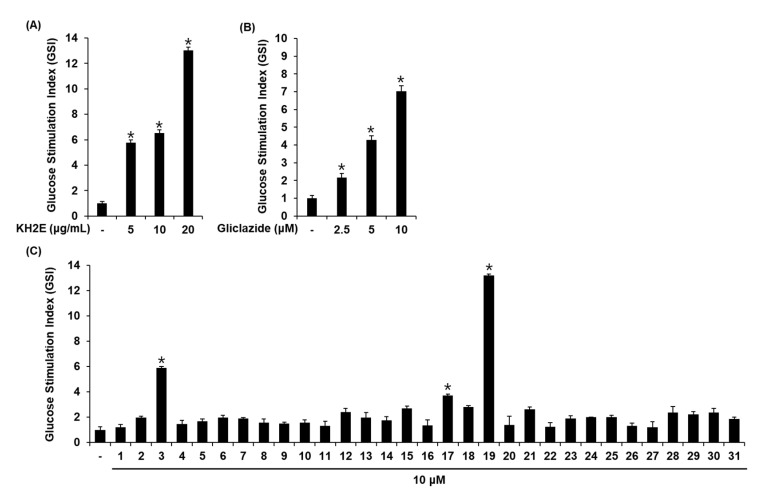
Effects of KH2E and compounds **1**–**31** on glucose-stimulated insulin secretion in INS-1 cells. Insulin secretion after 1 h treatment with 2.8 mM and 16.7 mM glucose in the presence or absence of (**A**) KH2E, (**B**) gliclazide, and (**C**) compounds **1**–**31**, as determined by the insulin secretion assay. Data represent the mean ± S.E.M., n = 3, * *p* < 0.05 compared with control.

We investigated whether KH2E, compounds **1**–**31**, and gliclazide (positive control) could increase GSIS in INS-1 cells. KH2E (2.5, 5, 10, 20 μg/mL) and gliclazide (1.25, 2.5, 5, 10 μM) did not induce cell death in INS-1 cells (Figure 4A,B). Compounds **1**–**31** at 10 μM did not induce cell death in INS-1 cells (Figure 4C). Next, we investigated whether KH2E and compounds **1**–**31** could increase GSIS. As shown in Figure 5, KH2E, isohydroxylomatin (**3**), (−)-marmesin (**17**), and marmesinin (**19**) increased the glucose-stimulated index (GSI), meaning GSIS. The GSI was calculated by dividing the insulin level at stimulating 16.7 mM glucose by the insulin level at 2.8 mM glucose. The GSI of the most effective marmesinin (**19**) was superior to that of gliclazide. These results indicated that marmesinin (**19**) enhanced GSIS without toxic effects on INS-1 cells.

### 3.3. Effect of Marmesinin (**19**) on GSIS

**Figure 6:**
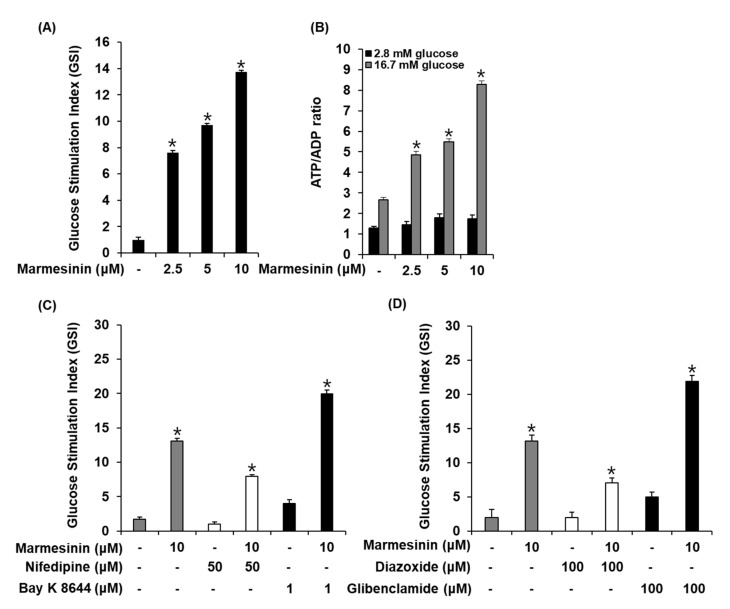
Effects of marmesinin (**19**) on glucose-stimulated insulin secretion in INS-1 cells. (**A**) Insulin secretion in INS-1 cells after 1 h incubation with basal (2.8 mM) and stimulant (16.7 mM) glucose concentrations in the presence or absence of marmesinin (**19**) by insulin secretion assay. (**B**) ATP/ADP ratio in INS-1 cells after 1 h incubation with basal (2.8 mM) and stimulant (16.7 mM) glucose concentrations in the presence or absence of marmesinin (**19**) by ADP/ATP ratio assay. (**C**) Insulin secretion in INS-1 cells after 1 h incubation with basal (2.8 mM) and stimulant (16.7 mM) glucose concentrations in the presence or absence of marmesinin (**19**), nifedipine (L-type Ca^2+^ channel blocker), and Bay K 8644 (L-type Ca^2+^ channel activator) by insulin secretion assay. (**D**) Insulin secretion in INS-1 cells after 1 h incubation with basal (2.8 mM) and stimulant (16.7 mM) concentrations of glucose in the presence or absence of marmesinin (**19**), diazoxide (K^+^ channel activator), and glibenclamide (K^+^ channel blocker) by the insulin secretion assay. Data represent the mean ± S.E.M., n = 3, * *p* < 0.05 compared with control.

We determined the effect of marmesinin (**19**) on the GSIS and ATP/ADP ratio. As shown in Figure 6A,B, marmesinin (**19**) increased the GSIS and glucose-dependent ATP/ADP ratio. Furthermore, we determined the efficiency of marmesinin (**19**) to modulate K^+^ and Ca^2+^ channels. As shown in Figure 6C,D, marmesinin-induced GSIS was enhanced by Bay K 8644 (L-type Ca^2+^ channel agonist) and glibenclamide (K^+^ channel blocker), while abrogated by nifedipine (L-type Ca^2+^ channel blocker) and diazoxide (K^+^ channel activator).

### 3.4. Effect of KH2E and Marmesinin (**19**) on the Protein Expression of P-IRS-2 (Ser731), IRS-2, PPARγ, and PDX-1

**Figure 7:**
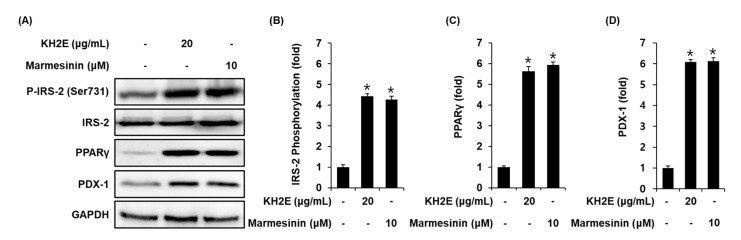
Effect of KH2E and marmesinin (**19**) on the protein expression levels of phospho-insulin receptor substrate-2 [P-IRS-2 (Ser731)], IRS-2, peroxisome proliferator-activated receptor γ (PPARγ), and pancreatic and duodenal homeobox 1 (PDX-1). (**A**) Protein expression levels of P-IRS-2 (Ser731), IRS-2, PPARγ, PDX-1, and GAPDH (glyceraldehyde 3-phosphate dehydrogenase) in INS-1 cells treated or untreated with 20 μg/mL KH2E and 10 μM marmesinin (**19**) for 24 h. (**B**–**D**) Each bar graph presents densitometric quantification of western blot bands. Data represent the mean ± S.E.M., n = 3, * *p* < 0.05 compared with the control.

Compared with untreated controls, INS-1 cells treated with 20 μg/mL KH2E and 10 μM marmesinin (**19**) showed increased protein expression of PPARγ, PDX-1, and phosphorylation levels of IRS-2 (Figure 7).

## 4. Discussion

Previous studies have reported that plants including *Rhizophora mucronata*, *Cassia glauca*, and *Urtica dentata* exhibit antidiabetic activity. These plants contain coumarin compounds and decrease blood glucose level in diabetic treated rats [51,52,53]. A previous review study of the anti-diabetic effect of coumarins reports the pharmacodynamics of simple coumarins, furanocoumarins, and pyranocoumarins, chemically classified based on the substitution of lactone and benzene rings in various experimental diabetes mellitus models [54]. In the present study, findings showed that KH2E, an extract from the roots of *A*. *reflexa*, significantly enhanced GSIS in INS-1 pancreatic β-cells. Thus, to find new active substances of KH2E, we isolated and identified 31 compounds (**1**–**31**) including 3 new compounds (**1**–**3**) from KH2E. Among the compounds **1**–**31**, three coumarins, isohydroxylomatin (**3**), (−)-marmesin (**17**), and marmesinin (**19**), increased GSIS. The biological activity of a new compound, isohydroxylomatin (**3**), has not yet been reported. Furocoumarin, (−)-marmesin (**17**, nodakenetin), isolated from *angelica decursiva,* has been reported to have anti-diabetic and anti-Alzheimer-related activities [55]. It has also been reported that (−)-marmesin (**17**) exhibits anticancer effects against human leukemia cells in vitro and in vivo [56]. On the other hand, marmesin, stereoisomer of **17**, isolated from *Aegle marmelos,* induces both pancreatic β cell regeneration and insulin secretion [57]. Marmesin has been reported to exert anti-cancer activities [58,59,60]. Further, it has been reported that marmesin directly stimulates glucose or acts like insulin to increase glucose utilization [61]. Marmesinin (**19**) isolated from *Angelica gigas* has been reported to exhibit neuroprotective and antiplasmodial effects [62,63]. Marmesinin (**19**) isolated from the bark of *Streblus indicus* has been reported to exhibit antimicrobial activity, and this compound isolated from the stem bark of *Zanthoxylum leprieurii* has been reported to exhibit antimycobacterial activity. [64,65]. Additionally, marmesinin (**19**) has been reported to significantly reduce lipidperoxide-induced myocardial damage in rats [66,67].

In the present study, marmesinin (**19**) was the most effective on GSIS assay. This effect was superior to treatment with gliclazide, which is often performed in patients with T2D. Marmesinin (**19**) is a furocoumarin glycoside. The effects of glycoside on insulin secretion have previously been reported in INS-1 cells and isolated mouse islets [68,69]. In addition, glycoside such as stevioside, rutin, and puerarin have been reported for antidiabetic activity [70].

In an additional experiment, treatment with marmesinin (**19**) resulted in increases in the ATP/ADP ratio. Glibenclamide and Bay K 8644 enhanced GSIS by marmesinin (**19**), whereas it was suppressed by nifedipine and diazoxide. Previous studies have shown that an increased ATP/ADP ratio is essential in the Ca2^+^ influx and closure of ATP-sensitive K^+^ (KATP) channels [71]. Diazoxide and nifedipine decrease insulin secretion, whereas glibenclamide and Bay K 8644 increase insulin secretion in pancreatic β-cells [72,73,74,75]. Taken together, our findings suggested that after treatment with marmesinin (**19**), the ability of the pancreatic β-cell to secrete insulin may be due to closure of ATP-sensitive K^+^ (KATP) channels, Ca^2+^ influx, and an increase in the ATP/ADP ratio. Furthermore, expression of PPARγ, PDX-1, and IRS-2 were increased and partly attributable to increased insulin secretion following treatment with marmesinin (**19**). Previous examples of literature have shown that PPARγ activation via full agonists increase GSIS in INS-1 cells [76]. It has been reported that PPAR-γ agonists can protect β cells from apoptosis and restore β cell functions, including GSIS [77]. It has also been described that IRS-2 knockout mice and PDX-1 knockout mice have been reported to display decreased GSIS [78,79]. Accumulating evidence suggests that GSIS from pancreatic β-cells requires an increase in protein expressions of PPARγ, IRS-2, and PDX-1. Consequently, our results show that KH2E can be considered as a potential anti-diabetic plant by increasing insulin secretion in pancreatic β-cells. Therefore, its anti-diabetic effect is attributed to the presence of marmesinin (**19**). Further preclinical investigations are required to fully understand the potency of marmesinin (**19**) as a possible antidiabetic agent.

## 5. Conclusions

Three new compounds (**1**–**3**) along with twenty-eight known compounds (**4**–**31**) were isolated from KH2E, the roots extract of *A*. *reflexa*. All the isolated compounds were evaluated for their anti-diabetic activity. The present study demonstrated that marmesinin (**19**) enhances GSIS in INS-1 cells. KH2E and marmesinin (**19**) increased phosphorylation levels of IRS-2 and activation of PPARγ and PDX-1, associated with pancreatic β-cell functions. Marmesinin (**19**) enhanced GSIS by shifting the intracellular ATP/ADP ratio and regulating K^+^ and Ca^2+^ channels. These findings promote potential application of marmesinin (**19**) toward the management of hyperglycemia in T2D, and future studies, including animal experiments, would greatly extend our understanding of the additional mechanisms of action of marmesinin (**19**).

## Figures and Tables

**Table 1:** ^1^H and ^13^C NMR data for new compounds **1**–**3**.

	1 ^a,c^		2 ^a,d^		3 ^b,c^	
Position	*δ*_C_, Type	*δ*_H_ Multi (*J* in Hz)	*δ*_C_, Type	*δ*_H_ Multi (*J* in Hz)	*δ*_C_, Type	*δ*_H_ Multi (*J* in Hz)
2	161.1, C		161.3, C		163.4, C	
3	112.9, CH_2_	6.24, d (9.9)	112.8, CH_2_	6.30, d (9.7)	112.5, CH	6.19, d (9.5)
4	139.3, CH	8.12, d (9.9),	139.6, CH	8.24, d (9.7),	146.5, CH	7.86, d (9.5)
5	148.6, C		148.9, C		130.5, CH	7.41, d (8.3)
6	113.3, C		114.1, C		108.1, CH	6.78, d (8.3)
7	158.1, C		158.1, C		165.8, C	
8	94.4, CH	7.14, s	94.5, CH	7.48, s	115.4, C	
9	152.6, C		152.6, C		152.7, C	
10	107.0, C		107.4, C		114.6, C	
2′	145.1, CH	7.61, d (2.2)	145.0, CH	7.61, d (2.0)	74.6, C	
3′	104.9, CH	7.01, d (2.2)	104.9, CH	6.95, d (2.0)	88.7, CH	5.02, dd (9.9, 8.0)
4′	-	-	-	-	68.2, CH_2_	3.32, dd (16.1, 9.9), 3.43, dd (16.1, 8.0)
5′	-	-	-	-	27.6, CH_2_	3.53, d (10.5), 3.72, d (10.5)
6′	-	-	-	-	19.7, CH_3_	1.21, s
1″	71.7, CH_2_	4.69, dd (10.2, 8.0), 4.85, dd (10.2, 2.6)	74.3, CH_2_	4.31, dd (9.9, 8.0), 4.48 dd (9.9, 3.0)	-	-
2″	77.4, CH	5.44, dd (8.0, 2.6)	77.4, CH	3.93, dd (8.0, 3.0)	-	-
3″	71.7, C		71.7, C		-	-
4″	26.5, CH_3_	1.38, s	26.5, CH_3_	1.21, s	-	-
5″	26.6, CH_3_	1.41, s	26.6, CH_3_	1.19, s	-	-
1′′′	126.5, C		131.8, C		-	-
2′′′	109.4, CH_2_	7.02, d (1.1)	128.3, CH	7.19, d (8.3)	-	-
3′′′	146.8, C		115.3, CH	6.80, d (8.2)	-	-
4′′′	148.4, C		155.4, C		-	-
5′′′	114.8, CH	6.94, d (8.2)	115.3, CH	6.80, d (8.2)	-	-
6′′′	123.3, CH	7.08, dd (8.2, 1.3)	128.3, CH	7.19, d (8.3)	-	-
7′′′	146.4, CH	7.67, d (15.9)	80.8, CH	4.34, t (5.7)	-	-
8′′′	114.2, CH	6.31, d (15.9)	66.3, CH_2_	3.52, dd (9.7, 4.9), 3.67 dd (9.7, 6.4)	-	-
9′′′	166.7, C		64.4, CH_2_	3.38, qd (9.2, 7.0), 3.45 qd (9.2, 7.0)	-	-
10′′′	-	-	15.2, CH_3_	1.18, t (7.0)	-	-
3′′′-OMe	56.0, CH_3_	3.95, s	-	-	-	-

^a^ Measured in CDCl_3_; ^b^ Measured in methanol-*d*_4_; ^c^ Recorded at 500 MHz (^1^H NMR) and 125 MHz (^13^C NMR); ^d^ Recorded at 600 MHz (^1^H NMR) and 150 MHz (^13^C NMR).

## Data Availability

Not applicable.

## Supplementary Materials

The following supporting information can be downloaded at: https://www.mdpi.com/article/10.3390/pharmaceutics15041239/s1, Figure S1: HRESIMS spectrum of compound **1**, Figure S2: ^1^H NMR spectrum (600 MHz, CDCl_3_) of compound **1**, Figure S3: ^13^C NMR spectrum (150 MHz, CDCl_3_) of compound **1**, Figure S4: HSQC spectrum of compound **1**, Figure S5: HMBC spectrum of compound **1**, Figure S6: COSY spectrum of compound **1**, Figure S7: NOESY spectrum of compound **1**, Figure S8: HRESIMS spectrum of compound **2**, Figure S9: ^1^H NMR spectrum (600 MHz, CDCl_3_) of compound **2**, Figure S10: ^13^C NMR spectrum (150 MHz, CDCl_3_) of compound **2**, Figure S11: HSQC spectrum of compound **2**, Figure S12: HMBC spectrum of compound **2**, Figure S13: COSY spectrum of compound **2**, Figure S14: HRESIMS spectrum of compound **3**, Figure S15: ^1^H NMR spectrum (600 MHz, methanol-*d*_4_) of compound **3**, Figure S16: ^13^C NMR spectrum (150 MHz, methanol-*d*_4_) of compound **3**, Figure S17: HSQC spectrum of compound **3**, Figure S18: HMBC spectrum of compound **3**, Figure S19: COSY spectrum of compound **3**, Figure S20: NOESY spectrum of compound **3**, Figure S21: ClustalW multiple ITS sequence alignment of four plant species (*A. reflexa*, *O. grosseserratum*, *N. forbesii*, and *N. incisum*) and herbal materials used in this work.
