# Supplementary material for: Chemical Constituents from the Roots of Angelica reflexa That Improve Glucose-Stimulated Insulin Secretion by Regulating Pancreatic β-Cell Metabolism
Source: Pharmaceutics. 2023 Apr 13;15(4):1239. doi: 10.3390/pharmaceutics15041239 (PMC10146581; doi:10.3390/pharmaceutics15041239)
Supplement: Supplementary file 1 [file pharmaceutics-15-01239-s001.zip › pharmaceutics-2177422-supplementary.pdf]

Supporting Information for

Chemical Constituents from the Roots of *Angelica reflexa* That Improve Glucose-Stimulated Insulin Secretion by Regu-lating Pancreatic β-Cell Metabolism

Hyo-Seon Kim[1,†], Dahae Lee[2,†], Young-Hye Seo[1,†], Seung-Mok Ryu[1], A-Yeong Lee[1], Byeong-Cheol Moon[1], Wook-Jin Kim[1], Ki-Sung Kang[2,*], Jun Lee[1,*]

[1]Herbal Medicine Resources Research Center, Korea Institute of Oriental Medicine (KIOM), Naju 58245, Republic of Korea

[2]Cooperative-Center of Natural Product Central Bank for Biological Evaluation, College of Korean Medicine, Gachon University, Seongnam 13120, Republic of Korea

Figure S1. HRESIMS spectrum of the new compound **1**.

Figure S2. ^{1}H NMR spectrum (600 MHz, $CDCl_3$) of the new compound **1**.

Figure S3. ^{13}C NMR spectrum (150 MHz, $CDCl_3$) of the new compound **1**.

Figure S4. HSQC spectrum of the new compound **1**.

Figure S5. HMBC spectrum of the new compound **1**.

Figure S6. COSY spectrum of the new compound **1**.

Figure S7. NOESY spectrum of the new compound **1**.

Figure S8. HRESIMS spectrum of the new compound **2**.

Figure S9. ^{1}H NMR spectrum (600 MHz, $CDCl_3$) of the new compound **2**.

Figure S10. ^{13}C NMR spectrum (150 MHz, $CDCl_3$) of the new compound **2**.

Figure S11. HSQC spectrum of the new compound **2**.

Figure S12. HMBC spectrum of the new compound **2**.

Figure S13. COSY spectrum of the new compound **2**.

Figure S14. HRESIMS spectrum of the new compound **3**.

Figure S15. ^{1}H NMR spectrum (600 MHz, methanol-d_4) of the new compound **3**.

Figure S16. ^{13}C NMR spectrum (150 MHz, methanol-d_4) of the new compound **3**.

Figure S17. HSQC spectrum of the new compound **3**.

Figure S18. HMBC spectrum of the new compound **3**.

Figure S19. COSY spectrum of the new compound **3**.

Figure S20. NOESY spectrum of the new compound **3**.

Figure S21. ClustalW multiple ITS sequence alignment of four plant species (*A. reflexa, O. grosseserratum*, *N. forbesii*, and *N. incisum*) and herbal materials used in this work.

S1. HRESIMS spectrum of the new compound **1**.

Single Mass Analysis
Tolerance = 30.0 PPM / DBE: min = -1.5, max = 50.0
Element prediction: Off
Number of isotope peaks used for i-FIT = 3
Monoisotopic Mass, Even Electron Ions
62 formula(e) evaluated with 1 results within limits (up to 20 closest results for each mass)
Elements Used:

Mass	Calc. Mass	mDa	PPM	DBE	Formula	i-FIT	i-FIT Norm	Fit Conf %	C	H	O
479.1347	479.1342	0.5	1.0	15.5	C26 H23 O9	85.4	n/a	n/a	26	23	9

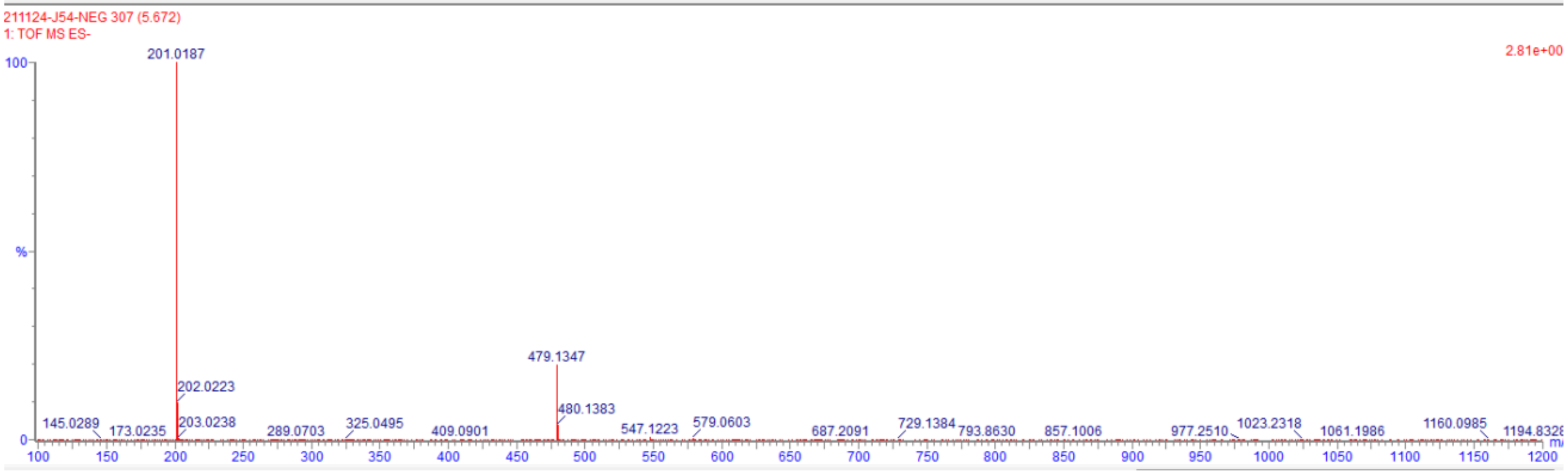

S2. ^{1}H NMR spectrum (600 MHz, $CDCl_3$) of the new compound **1**.

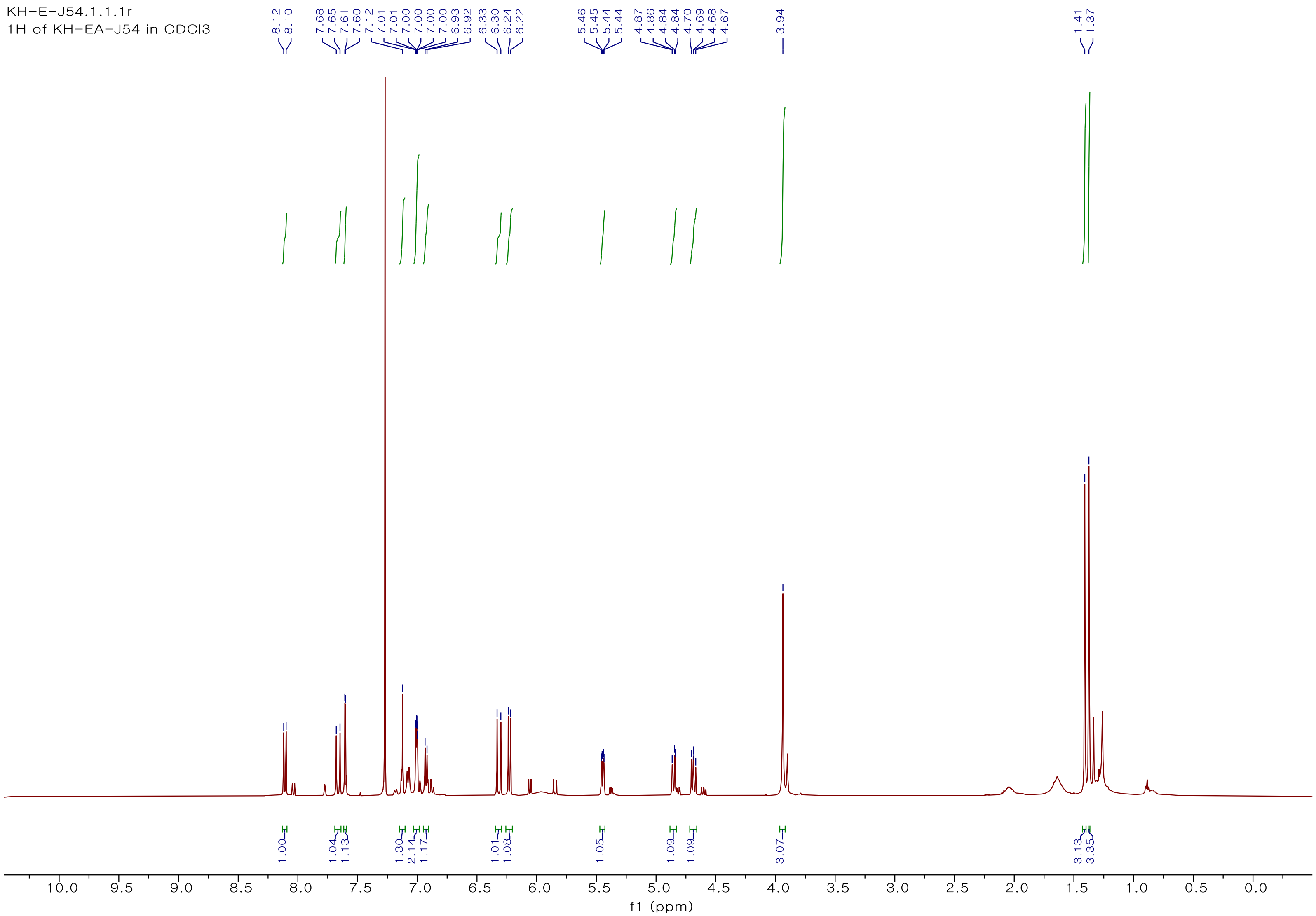

S3. ^{13}C NMR spectrum (150 MHz, $CDCl_3$) of the new compound **1**.

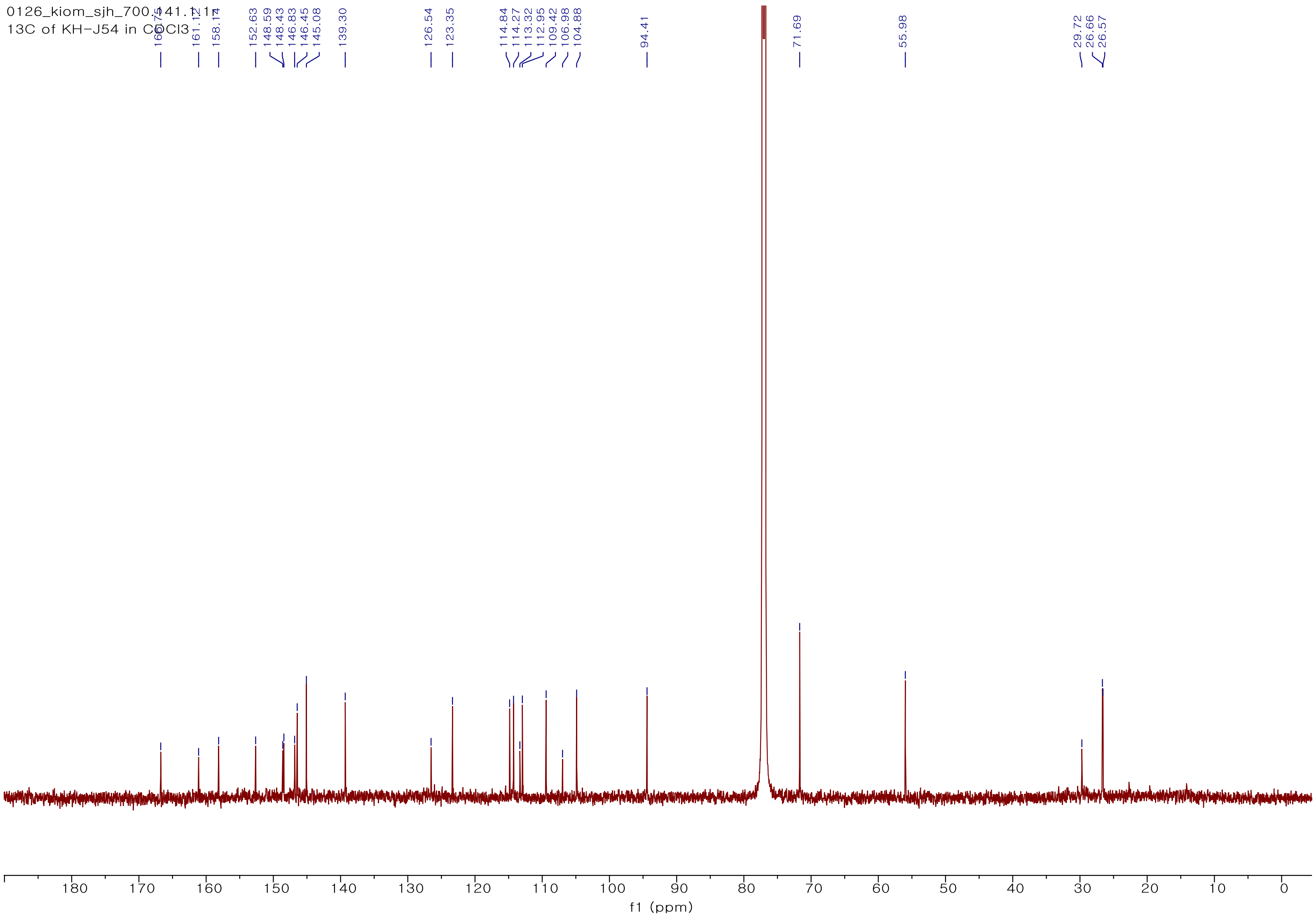

S4. HSQC spectrum of the new compound **1**.

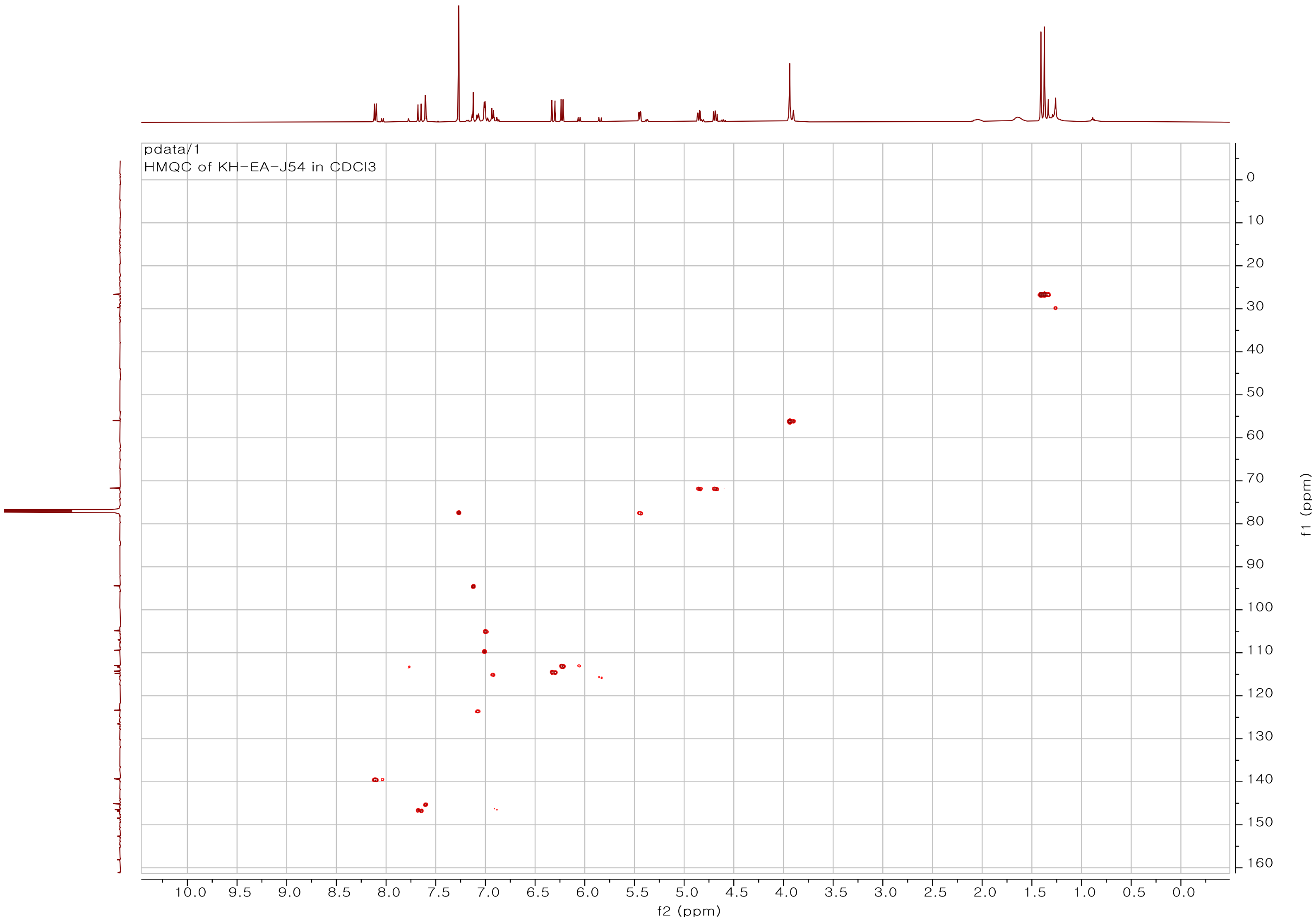

S5. HMBC spectrum of the new compound **1**.

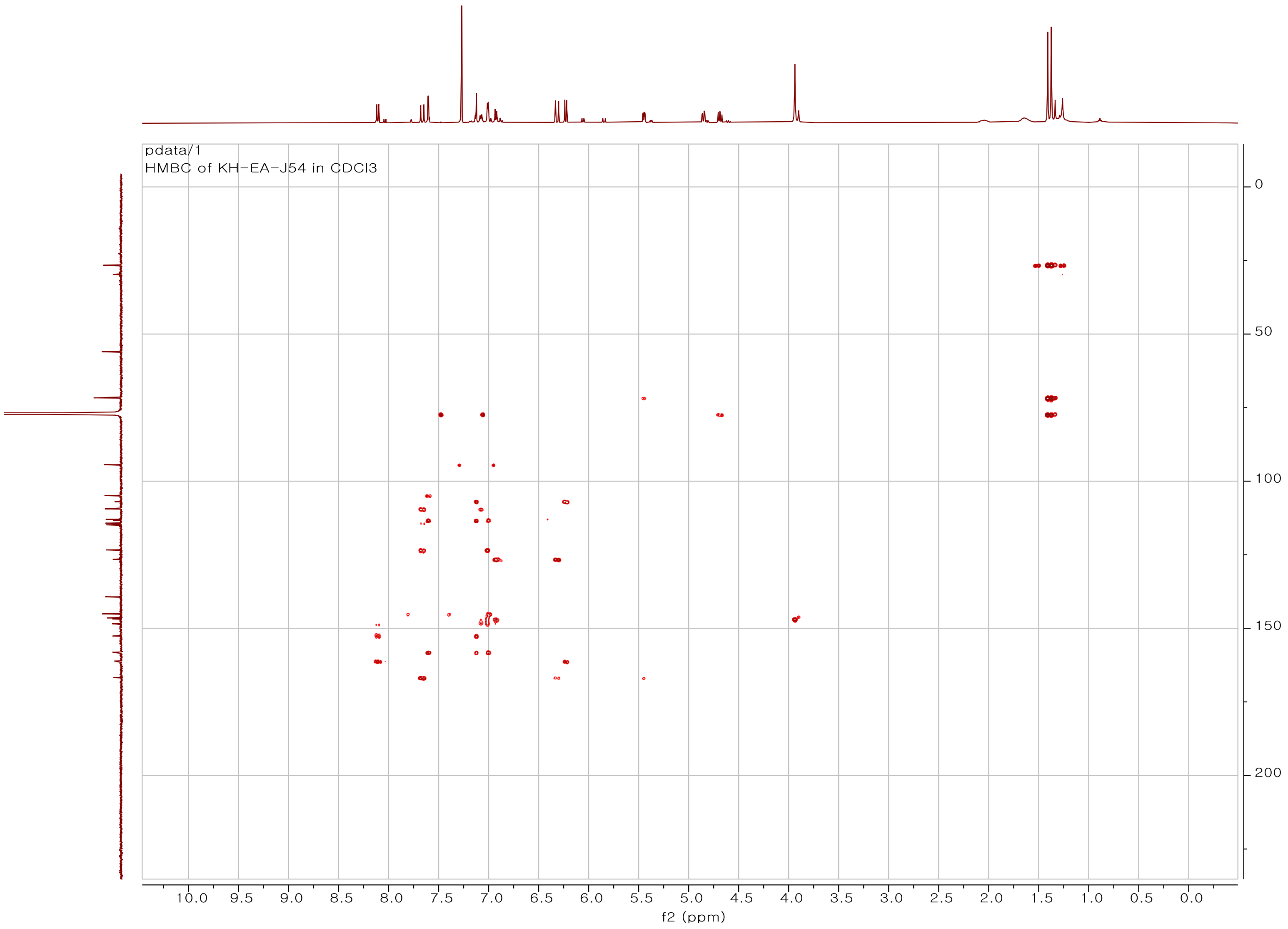

S6. COSY spectrum of the new compound **1**.

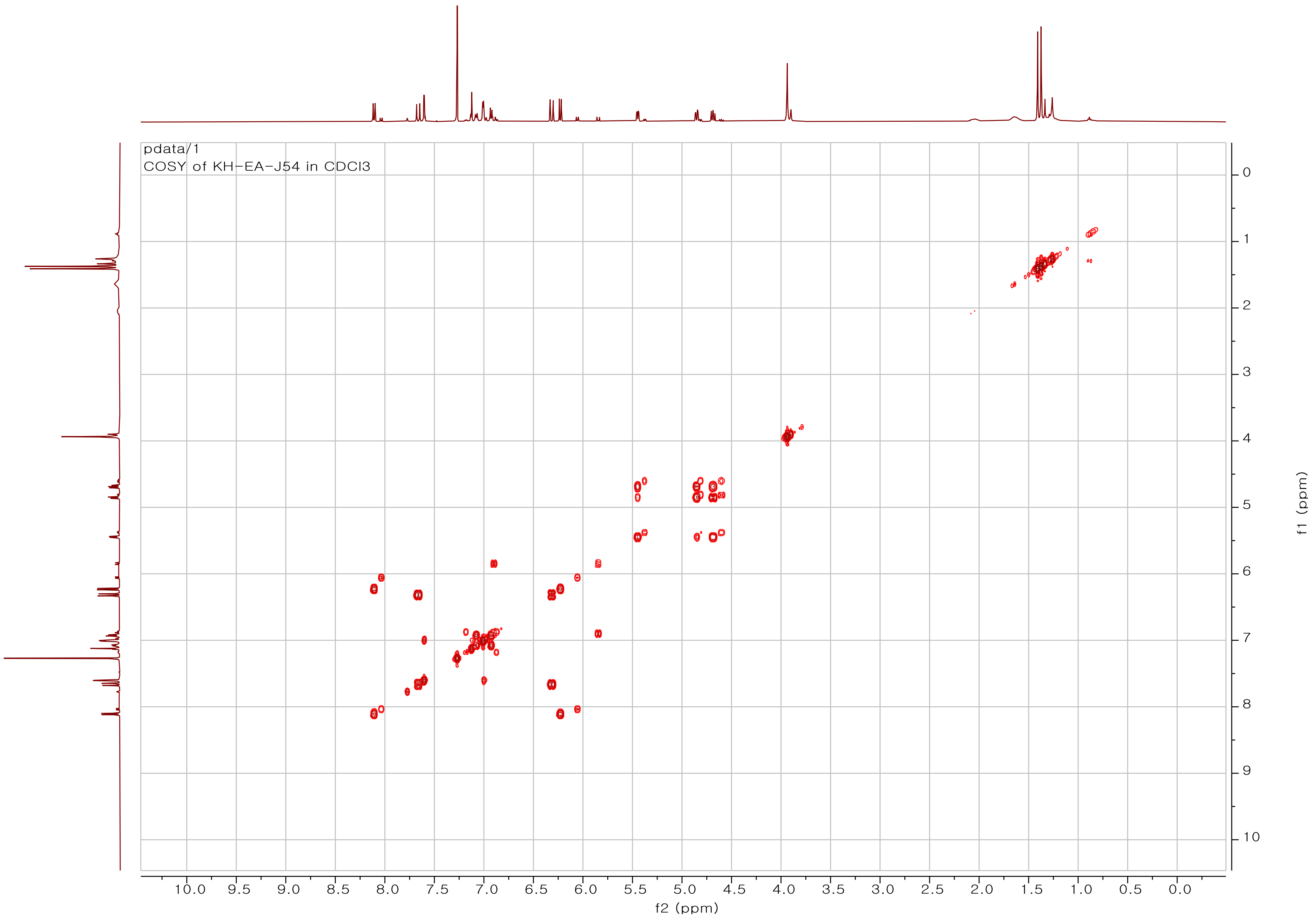

S7. NOESY spectrum of the new compound **1**.

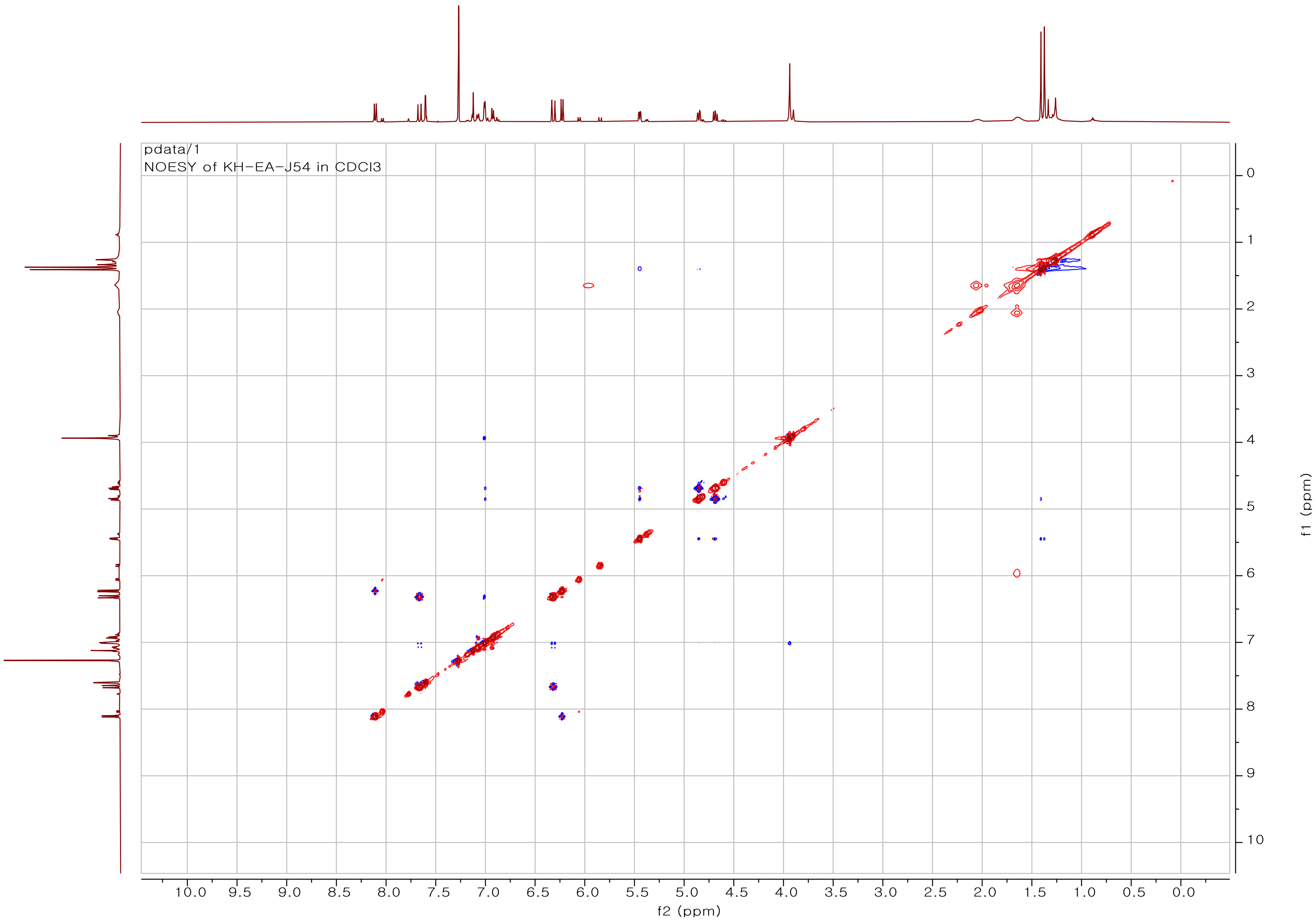

S8. HRESIMS spectrum of the new compound **2**.

Single Mass Analysis
Tolerance = 50.0 PPM / DBE: min = -1.5, max = 50.0
Element prediction: Off
Number of isotope peaks used for i-FIT = 3
Monoisotopic Mass, Even Electron Ions
46 formula(e) evaluated with 4 results within limits (up to 20 closest results for each mass)
Elements Used:

Mass	Calc. Mass	mDa	PPM	DBE	Formula	i-FIT	i-FIT Norm	Fit Conf %	C	H	O
467.1737	467.1706	3.1	6.6	13.5	C26 H27 O8	115.4	0.643	52.56	26	27	8
	467.1858	-12.1	-25.9	17.5	C30 H27 O5	115.9	1.124	32.50	30	27	5
	467.1917	-18.0	-38.5	8.5	C23 H31 O10	117.3	2.525	8.01	23	31	10
	467.1553	18.4	39.4	9.5	C22 H27 O11	117.4	2.670	6.93	22	27	11

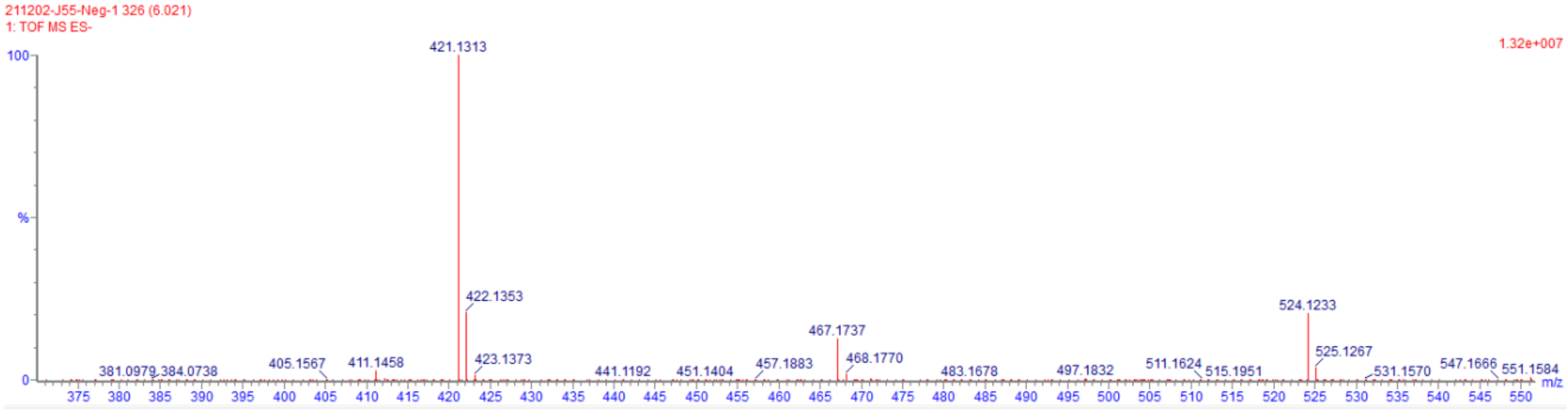

S9. ^{1}H NMR spectrum (600 MHz, $CDCl_3$) of the new compound **2**.

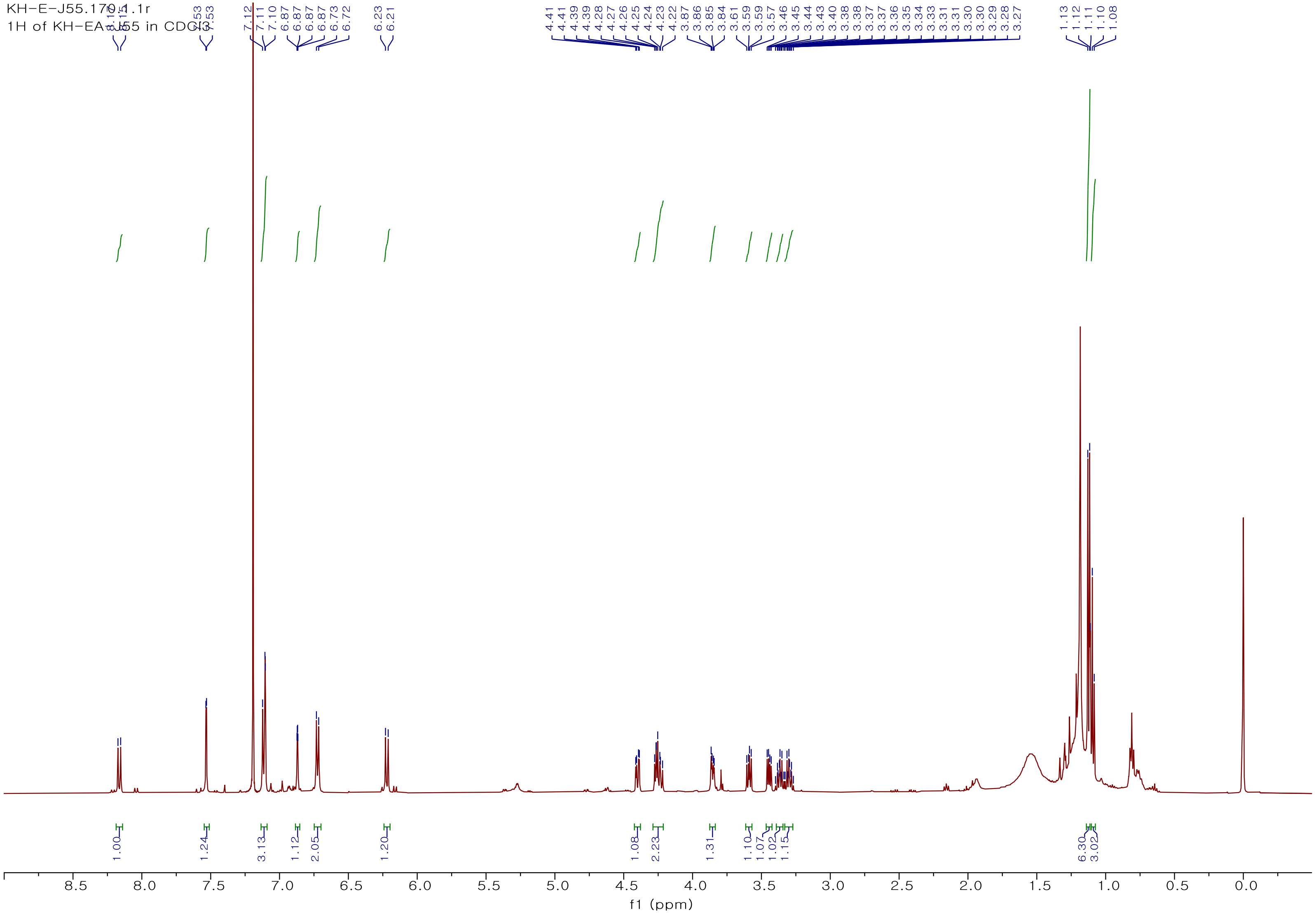

S10. ^{13}C NMR spectrum (150 MHz, $CDCl_3$) of the new compound **2**.

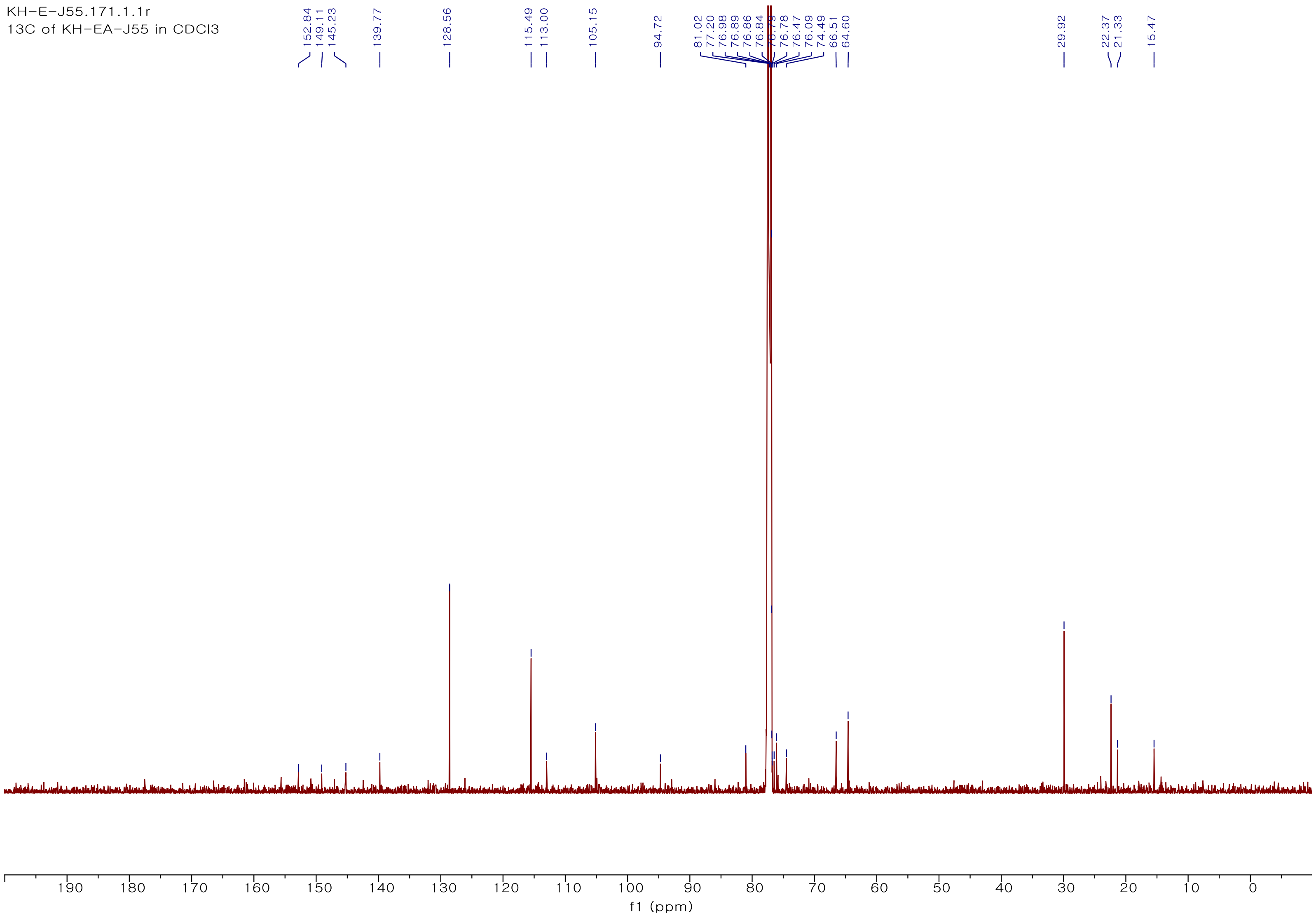

S11. HSQC spectrum of the new compound **2**.

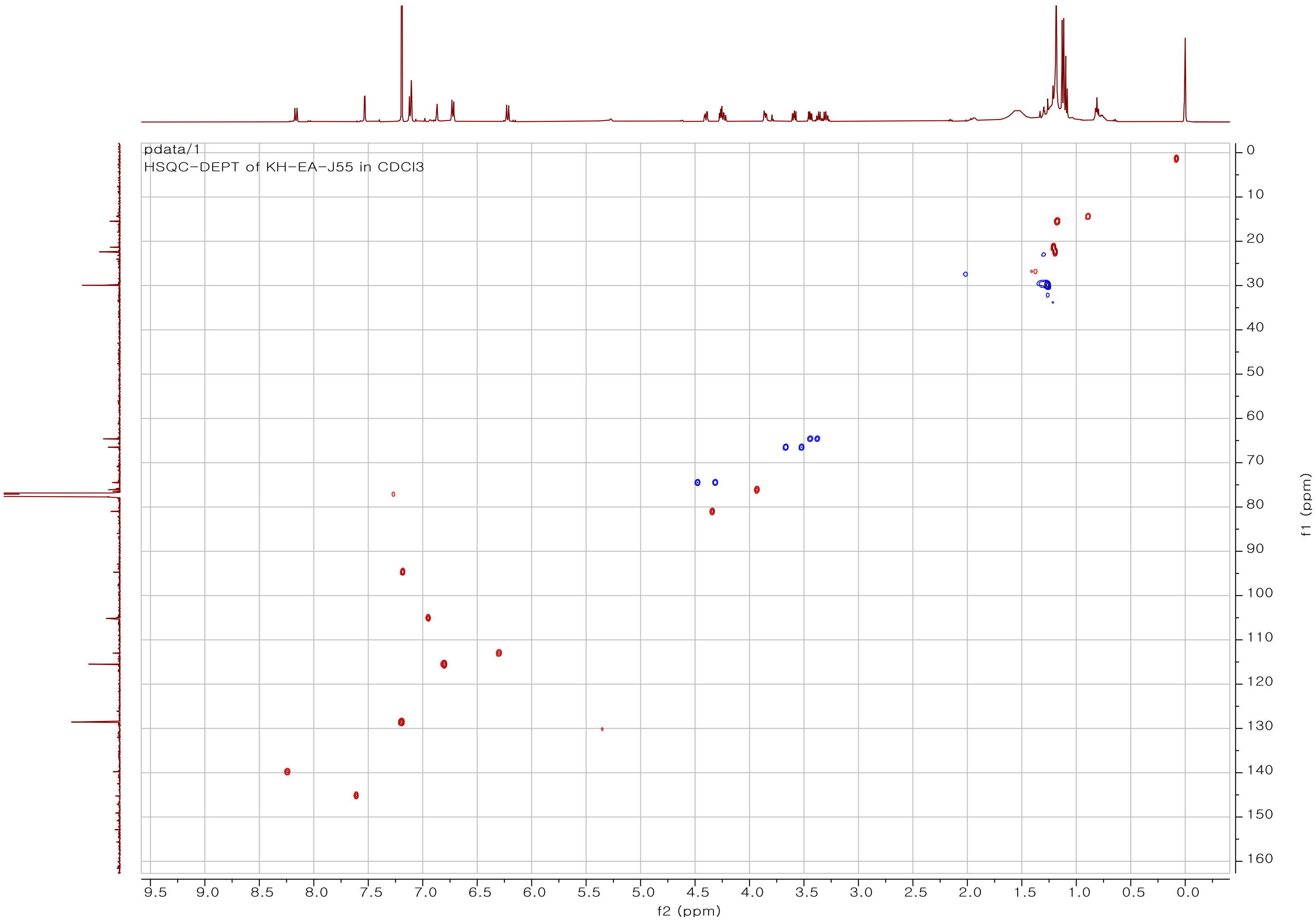

S12. HMBC spectrum of the new compound **2**.

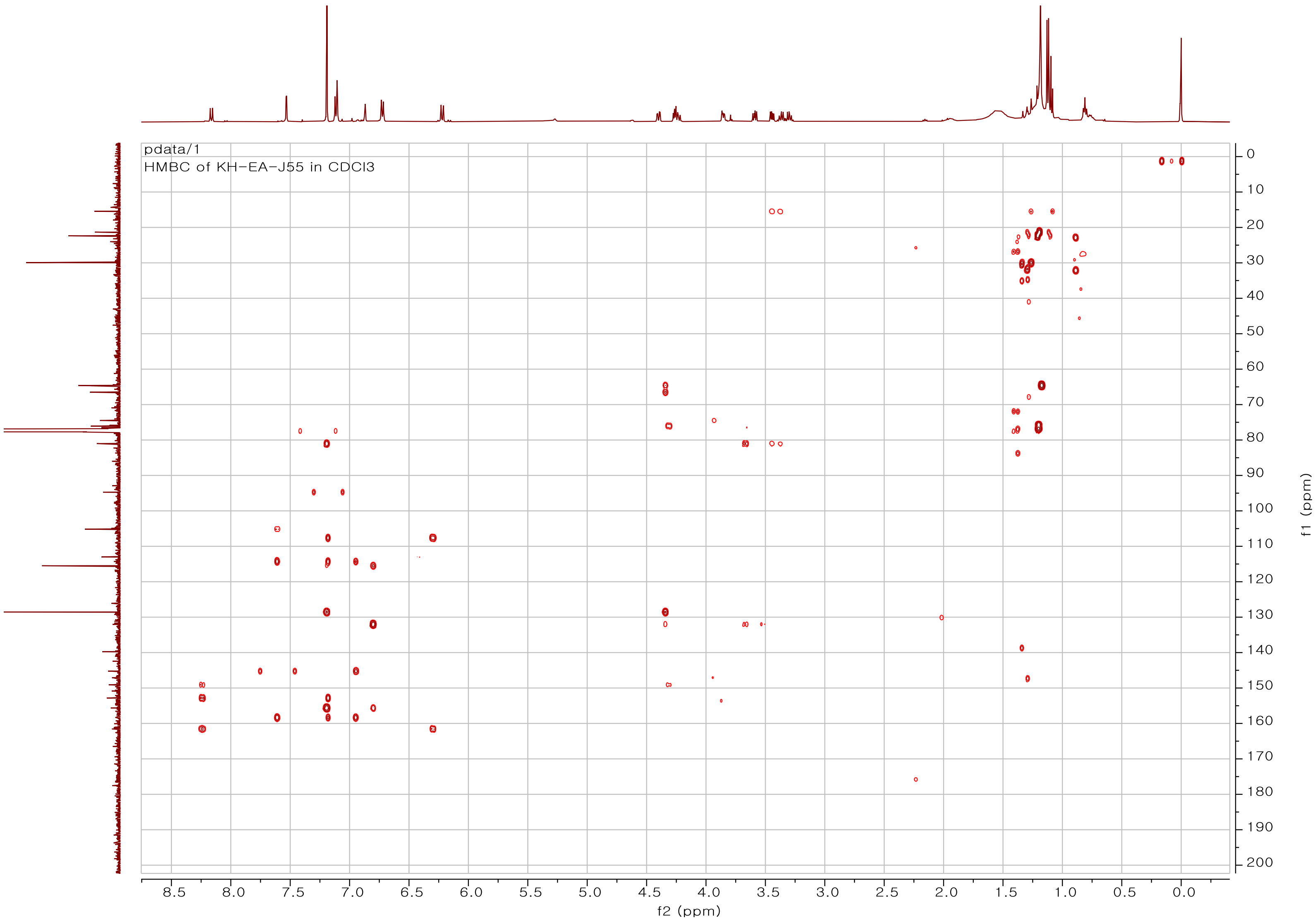

S13. COSY spectrum of the new compound **2**.

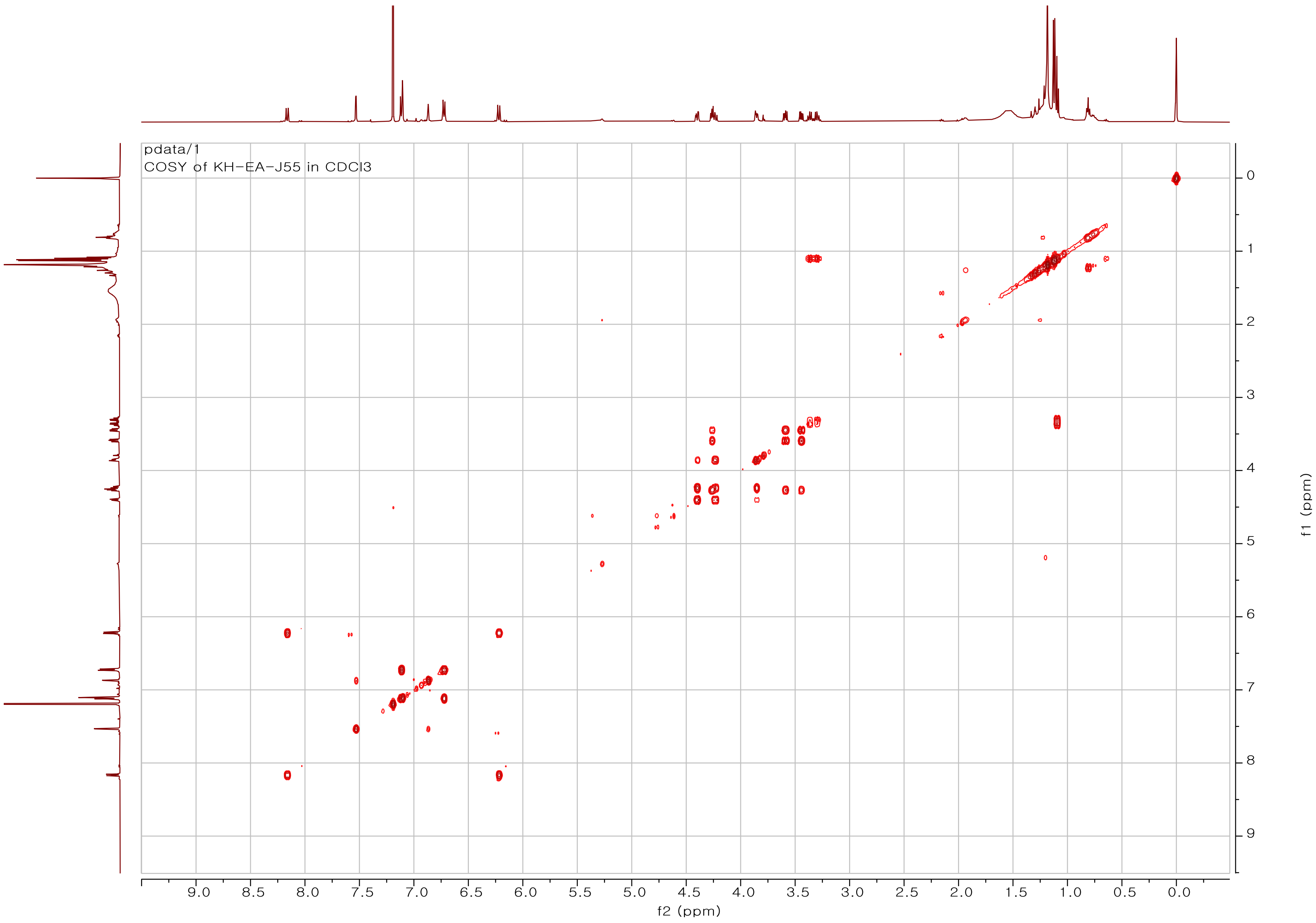

S14. HRESIMS spectrum of the new compound **3**.

Single Mass Analysis
Tolerance = 50.0 PPM / DBE: min = -1.5, max = 50.0
Element prediction: Off
Number of isotope peaks used for i-FIT = 3
Monoisotopic Mass, Even Electron Ions
47 formula(e) evaluated with 3 results within limits (up to 20 closest results for each mass)
Elements Used:

Mass	Calc. Mass	mDa	PPM	DBE	Formula	i-FIT	i-FIT Norm	Fit Conf %	C	H	O
263.0905	263.0919	-1.4	-5.3	7.5	C14 H15 O5	1188.2	0.012	98.82	14	15	5
	263.0861	4.4	16.7	16.5	C21 H11	1192.7	4.451	1.17	21	11	
	263.0978	-7.3	-27.7	-1.5	C7 H19 O10	1197.1	8.887	0.01	7	19	10

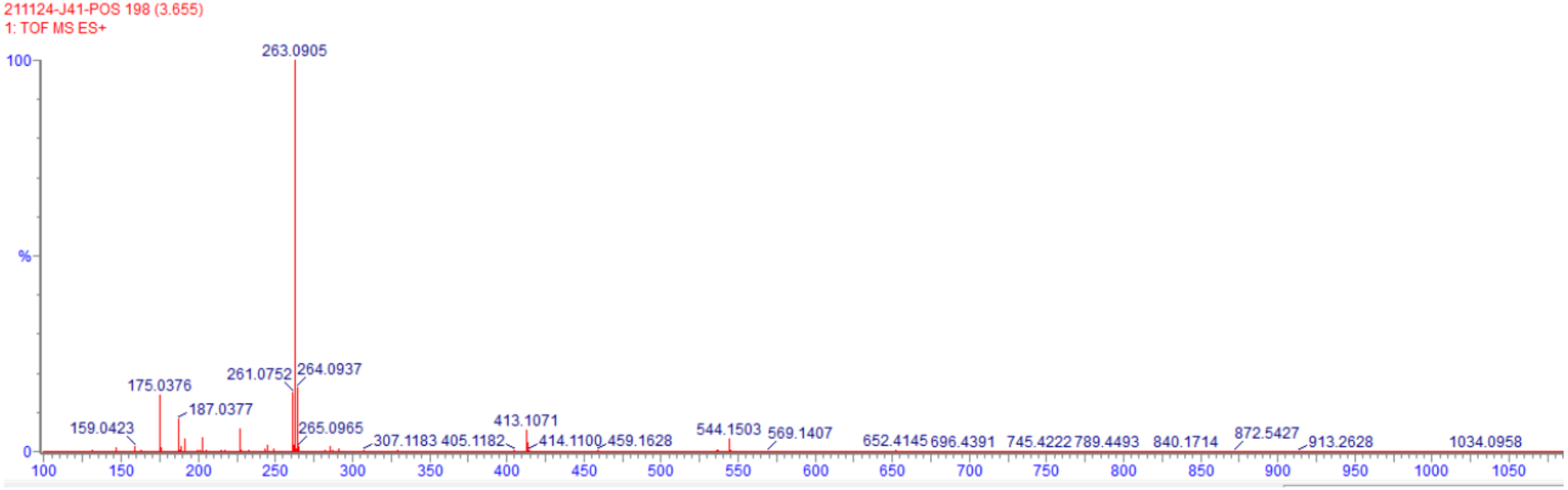

S15. ^{1}H NMR spectrum (600 MHz, methanol-d_4) of the new compound **3**.

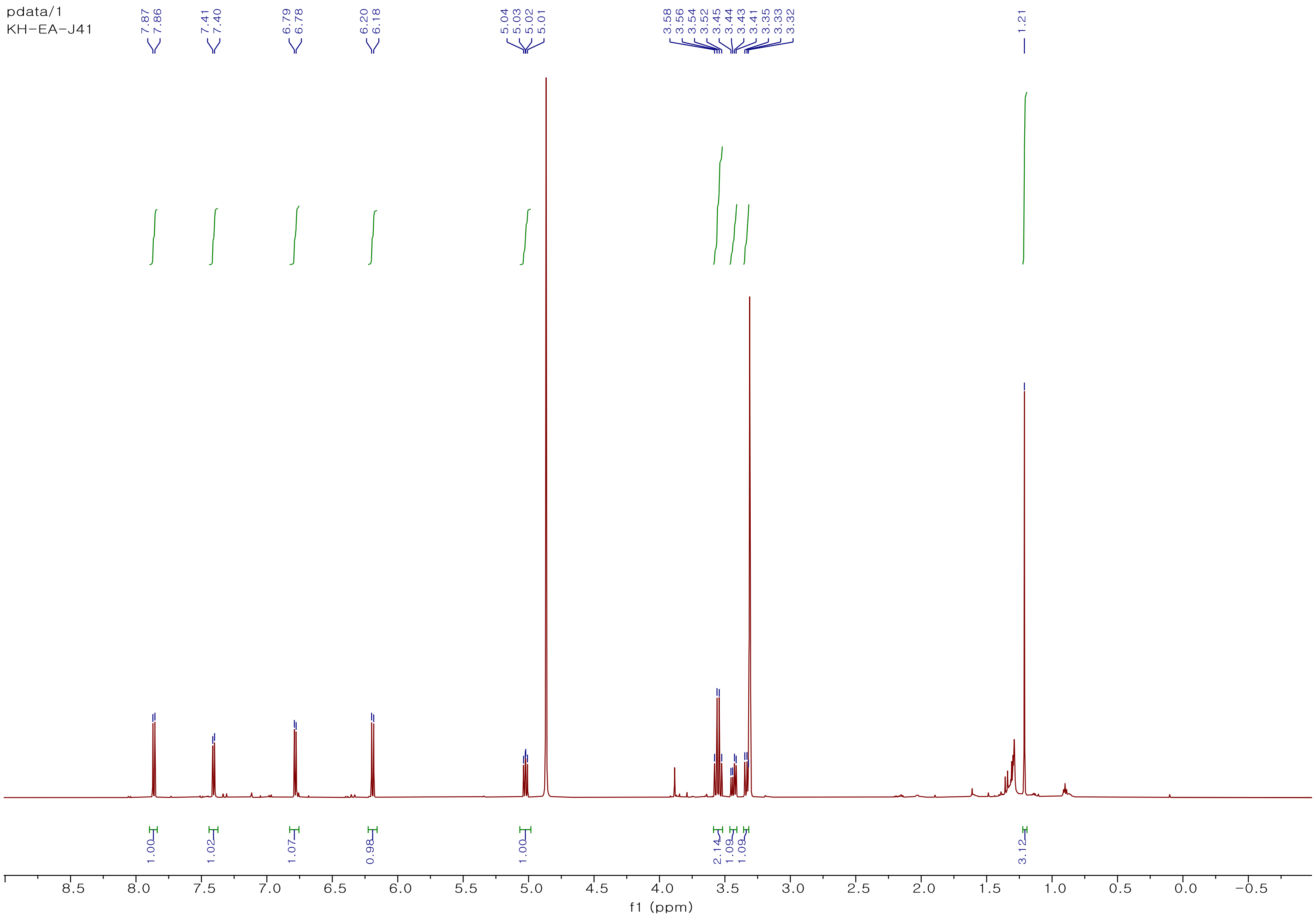

S16. ^{13}C NMR spectrum (150 MHz, methanol-d_4) of the new compound **3**.

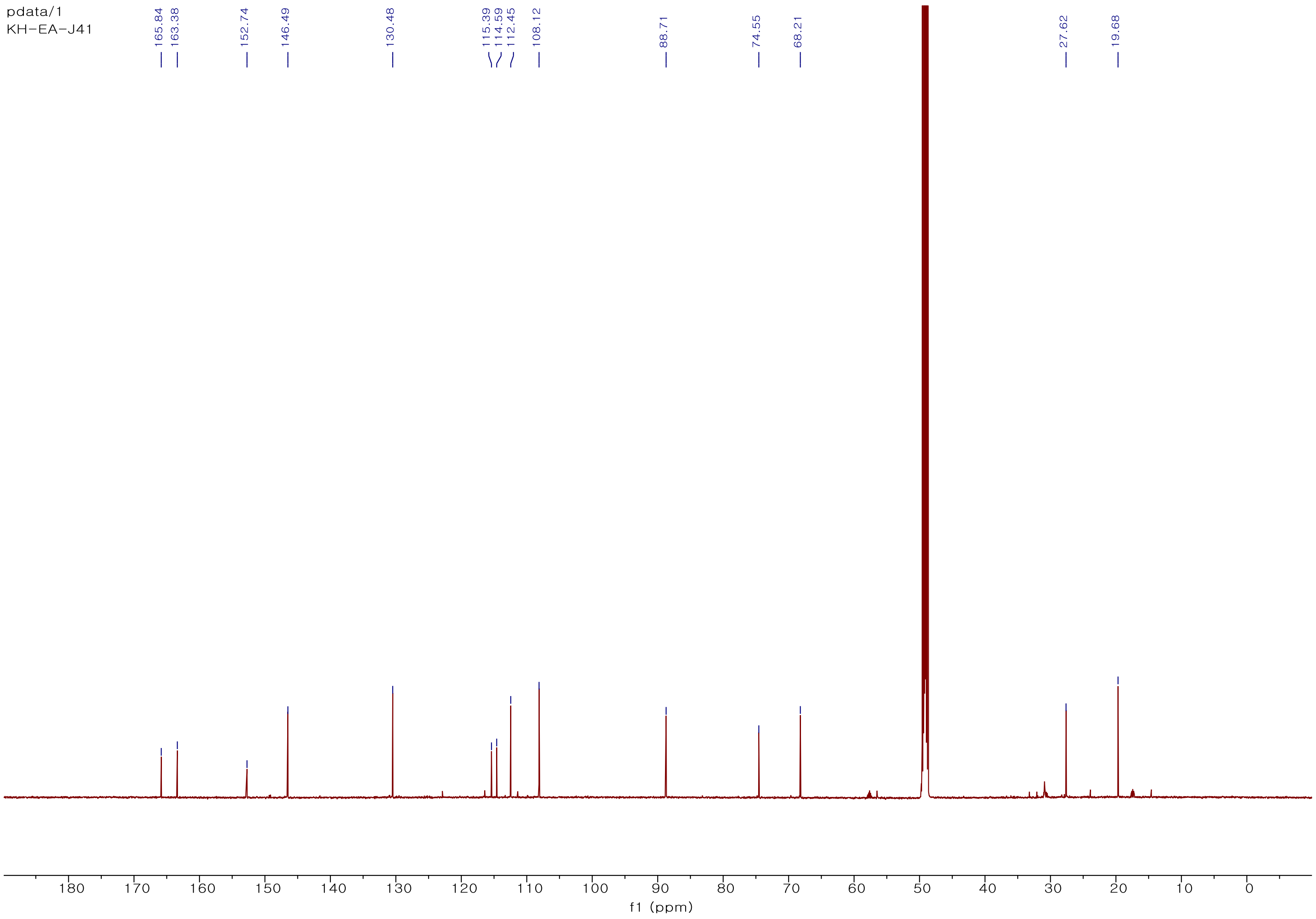

S17. HSQC spectrum of the new compound **3**.

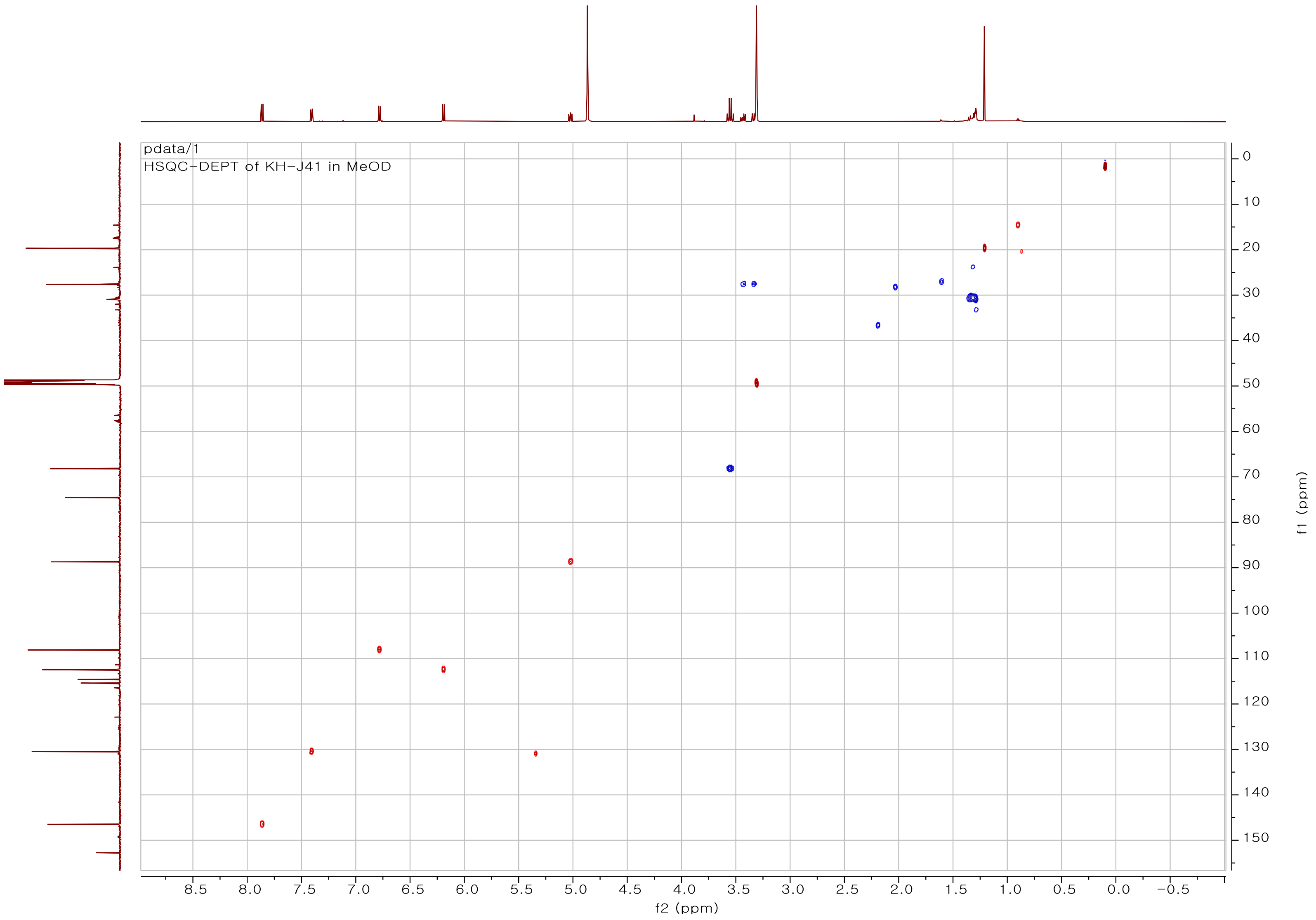

S18. HMBC spectrum of the new compound **3**.

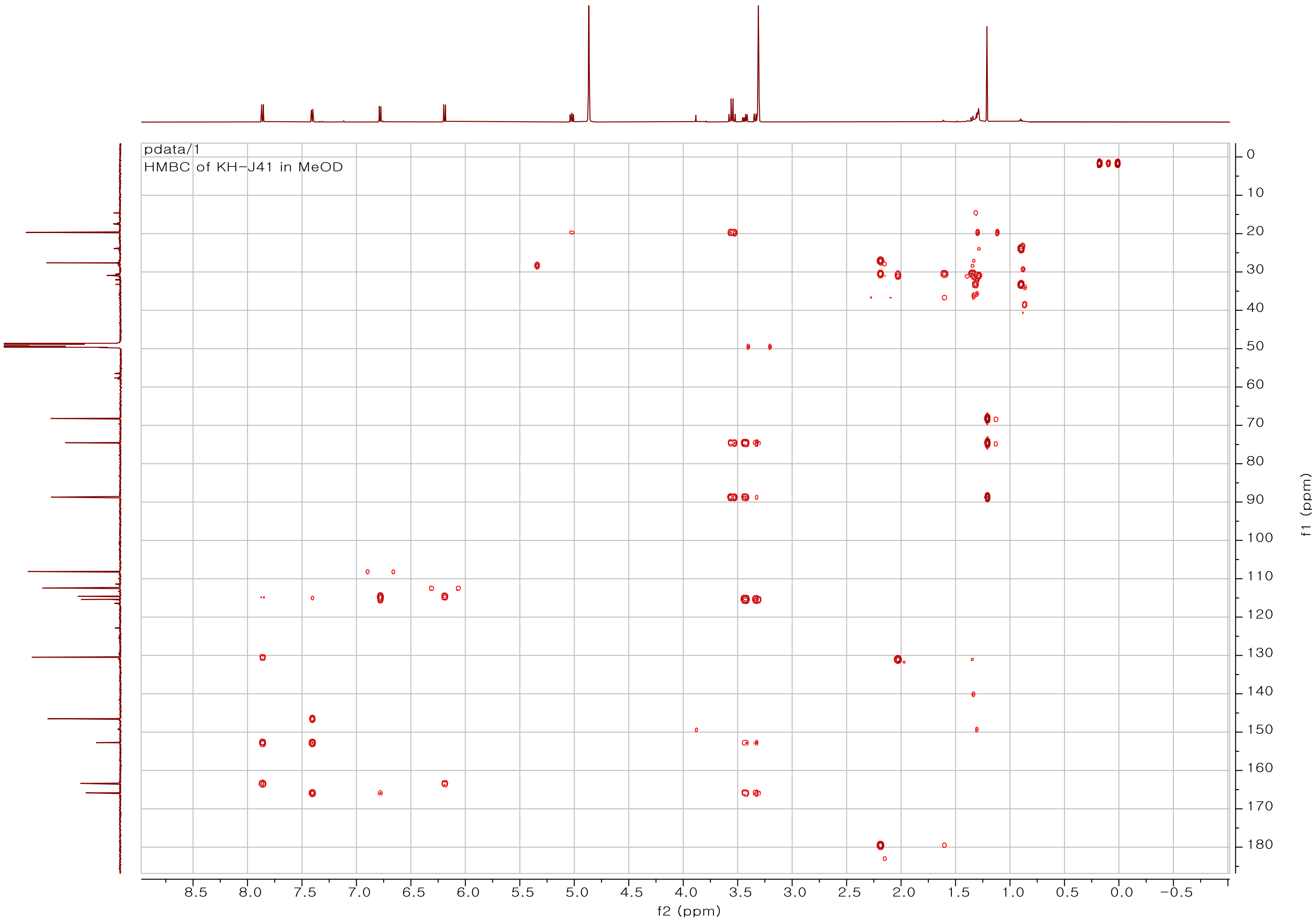

S19. COSY spectrum of the new compound **3**.

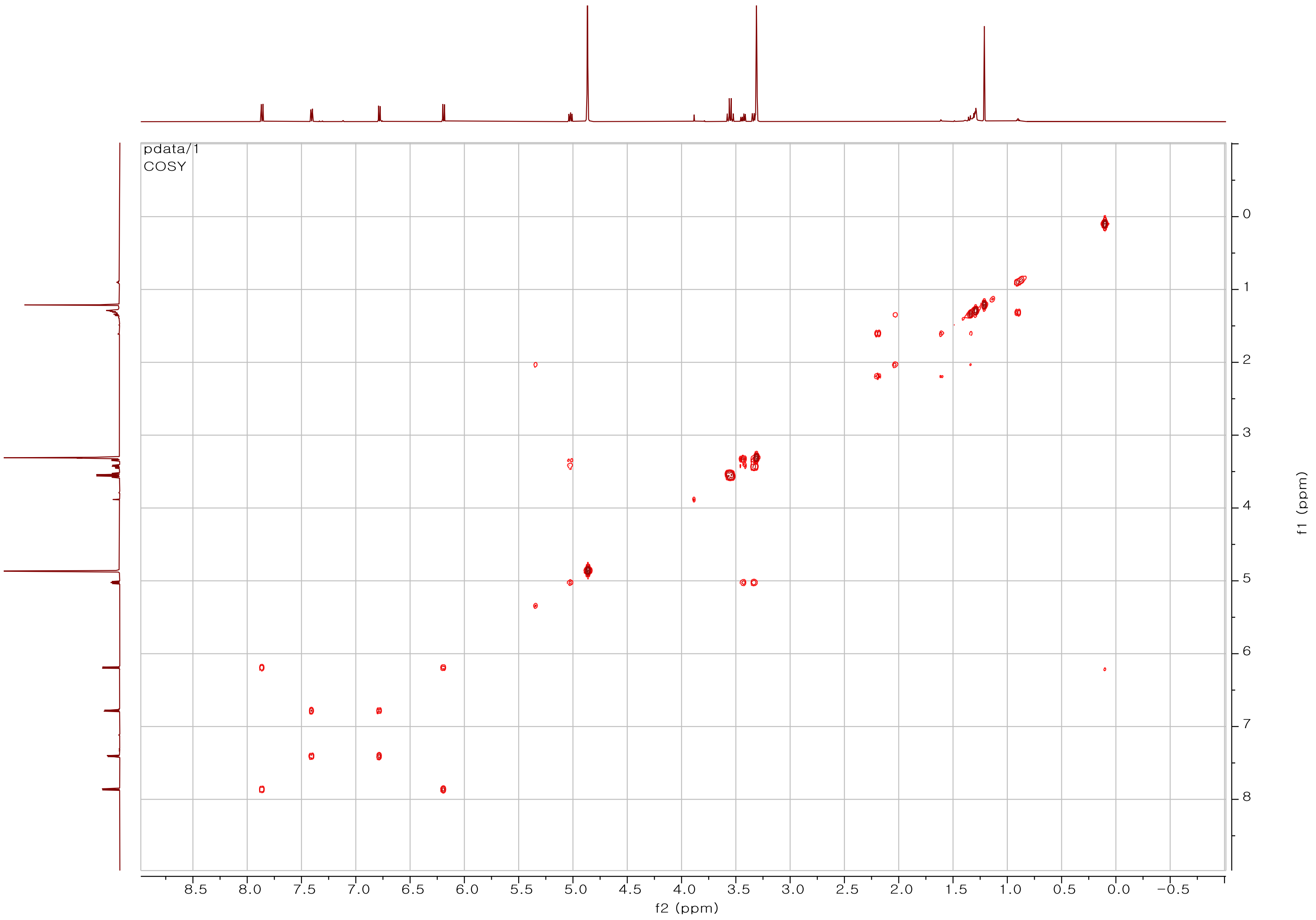

S20. NOESY spectrum of the new compound **3**.

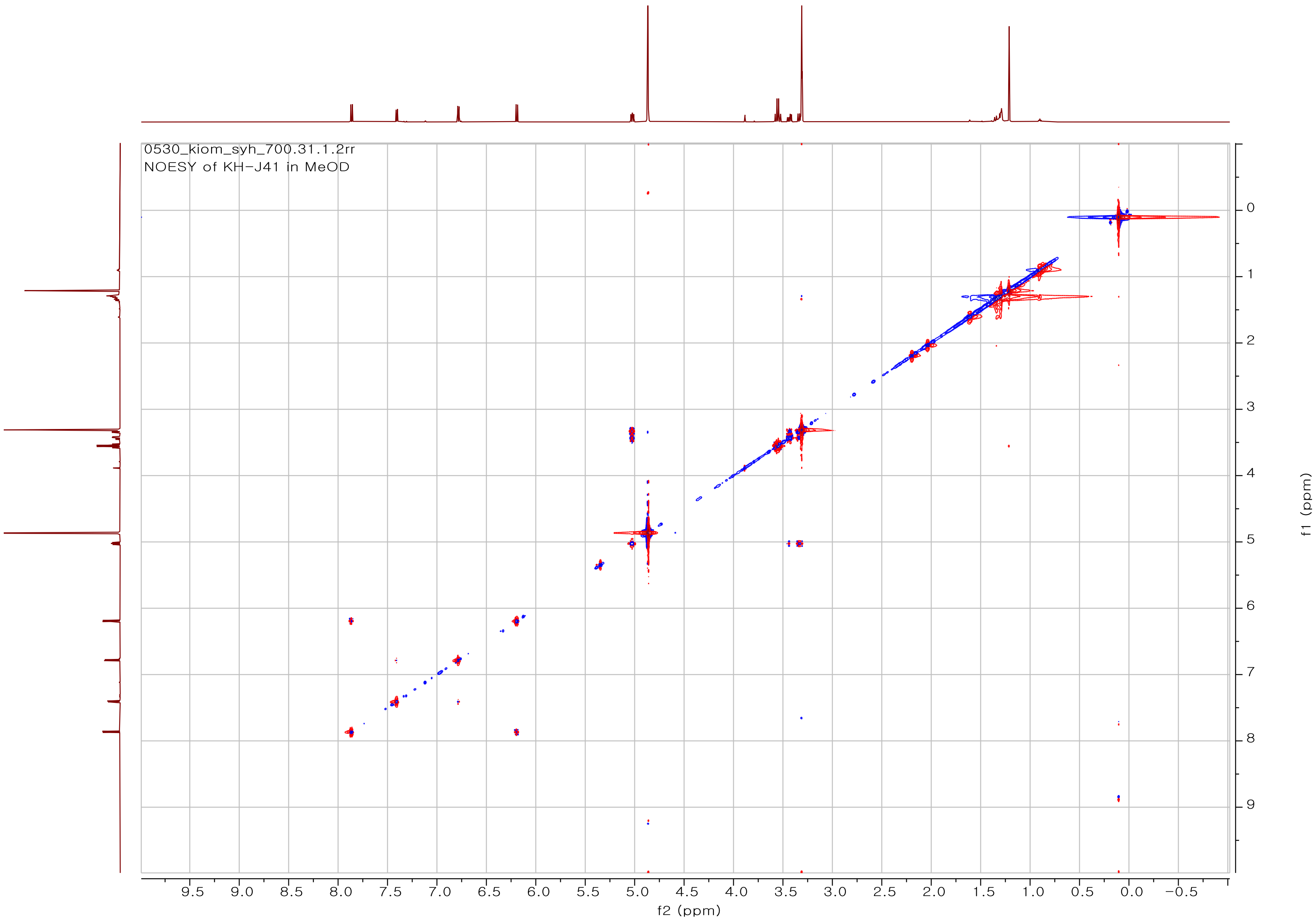

S21. ClustalW multiple ITS sequence alignment of four plant species (*A. reflexa*, *O. grosseserratum*, *N. forbesii*, and *N. incisum*) and herbal materials used in this work*.

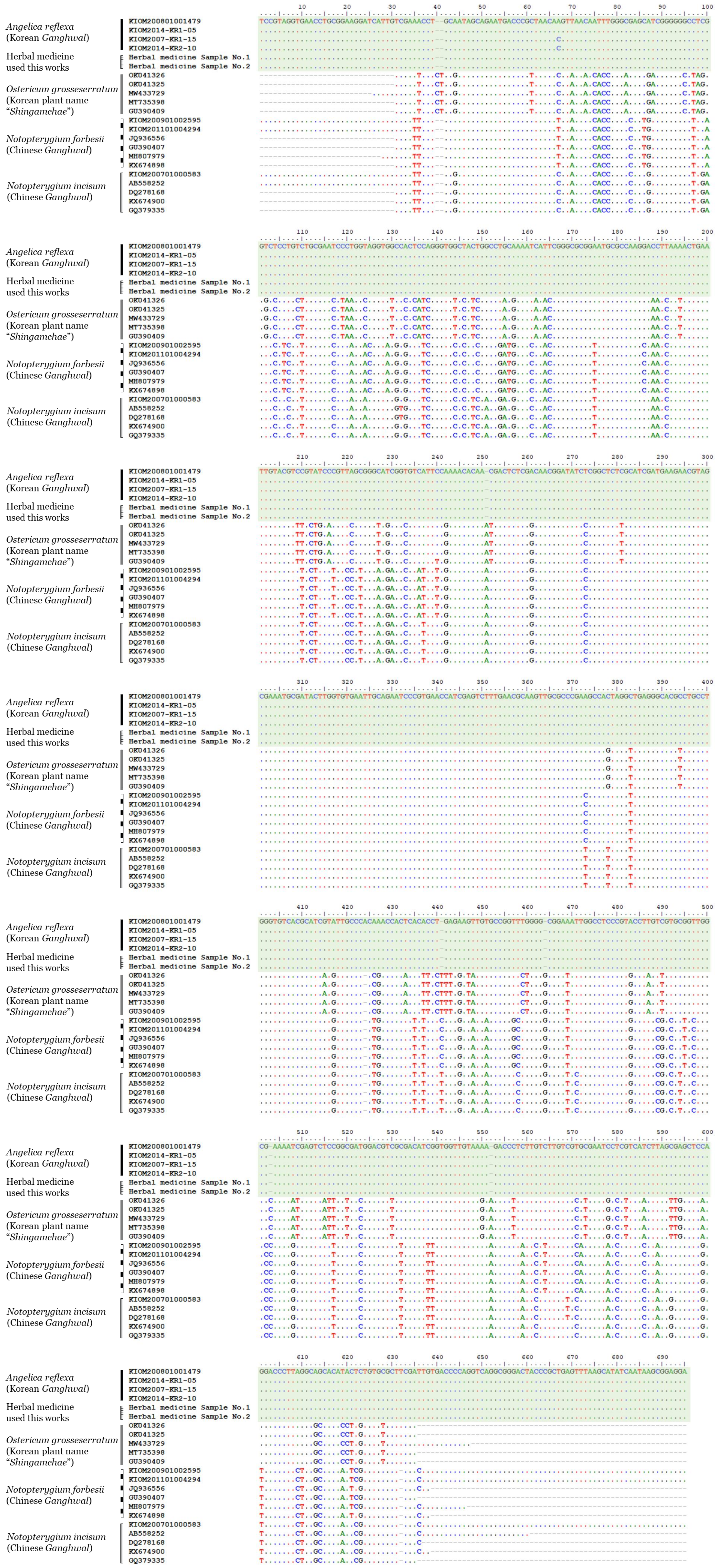

*ITS sequences of seven plant samples (KIOM200801001479, KIOM2014-KR1-05, KIOM2007-KR1-15, KIOM2014-KR2-10, KIOM200901002595, KIOM201101004294, and KIOM200701000583) and two herbal materials were obtained by the PCR amplification and sequencing for this work. The other thirteen ITS sequences were used registered in the GenBank database (OK041326, OK041325, MW433729, MT735398, and GU390409 for *O. grosseserratum*; JQ936556, GU390407, MH807979, and KX674898 for *N. forbesii*; AB558252, DQ278168, KX674900, and GQ379335 for *N. incisum*). Green box indicates the aligned ITS sequences of *A. reflexa* and two plant samples used in this study. Dots(·) indicate the identical sequences with KIOM200801001479 of *A. reflexa*, and dash(-) represents gaps introduced to maximize alignment.
